# Crystal structures and Hirshfeld surface analyses of methyl (2*Z*)-(4-bromo­phen­yl)[2-(4-methyl­phen­yl)hydrazinyl­idene]acetate, methyl (2*Z*)-(4-bromophen­yl)[2-(3,5-di­methyl­phen­yl)hydrazinyl­idene]acetate, methyl (2*Z*)-[2-(4-meth­oxy­phen­yl)hydrazinyl­idene](3-nitro­phen­yl)acetate, methyl (2*E*)-(4-chlorophen­yl)(2-phenyl­hydrazinyl­idene)acetate and methyl (2*Z*)-[2-(4-bromo­phen­yl)hydrazinylidene](4-chloro­phen­yl)acetate

**DOI:** 10.1107/S2056989025002051

**Published:** 2025-03-14

**Authors:** Namiq Q. Shikhaliyev, Aliyar A. Babazade, Gulnar T. Atakishiyeva, Irada M. Shikhaliyeva, Abel M. Maharramov, Victor N. Khrustalev, Zeliha Atioğlu, Mehmet Akkurt, Ajaya Bhattarai

**Affiliations:** a Department of Chemical Engineering, Baku Engineering University, Khirdalan City, 120 AZ0101 Hasan Aliyev Street, Baku, Azerbaijan; b Organic Chemistry Department, Baku State University, Z. Khalilov str. 23, AZ 1148 Baku, Azerbaijan; c Peoples’ Friendship University of Russia (RUDN University) Miklukho-Maklay St 6 Moscow 117198 Russian Federation; d N. D. Zelinsky Institute of Organic Chemistry RAS, Leninsky Prosp. 47, Moscow, 119991, Russian Federation; e Department of Aircraft Electrics and Electronics, School of Applied Sciences, Cappadocia University, Mustafapaşa, 50420 Ürgüp, Nevşehir, Türkiye; f Department of Physics, Faculty of Sciences, Erciyes University, 38039 Kayseri, Türkiye; g Department of Chemistry, M.M.A.M.C (Tribhuvan University) Biratnagar, Nepal; Vienna University of Technology, Austria

**Keywords:** crystal structure, esters, hydrogen bonds, C—H⋯π inter­actions, configuration.

## Abstract

Mol­ecules of the title compounds, methyl (*Z*)-2-(4-bromo­phen­yl)-2-[2-(4-methyl­phen­yl)hydrazin-1-yl­idene]acetate, C_16_H_15_BrN_2_O_*2*_, (**1**), methyl (*Z*)-2-(4-bromo­phen­yl)-2-[2-(3,5-di­methyl­phen­yl)hydrazin-1-yl­idene]acetate, C_17_H_17_BrN_2_O_2_, (**2**), methyl (*Z*)-2-[2-(4-meth­oxy­phen­yl)hydrazin-1-yl­idene]-2-(3-nitro­phen­yl)acetate, C_16_H_15_N_3_O_5_, (**3**), and methyl (*Z*)-2-[2-(4-bromo­phen­yl)hydrazin-1-yl­idene]-2-(4-chloro­phen­yl)acetate, C_15_H_12_BrClN_2_O_2_, (**5**), adopt a *Z* configuration with respect to the central C=N bond, while methyl (*E*)-2-(4-chloro­phen­yl)-2-(2-phenyl­hydrazin-1-yl­idene)acetate, C_15_H_13_ClN_2_O_2_, (**4**), adopts an *E* configuration. The atoms of the phenyl ring of the bromo­phenyl group of (**1**) are disordered over two sets of sites with equal occupancies. In the crystal structure of (**1**), mol­ecules connected by C—H⋯N hydrogen bonds are further linked by C—H⋯π inter­actions, forming ribbons parallel to [010]. In (**2**), pairs of mol­ecules are linked by C—H⋯π inter­actions parallel to [100]. In (**3**), C—H⋯O hydrogen bonds form ribbons parallel to [010], while in (**4**), the mol­ecules are bonded together by C—H⋯N, C—H⋯Cl, C—H⋯O and C—H⋯π inter­actions parallel to [010]. In (**5**), C—H⋯Br, C—H⋯O and C—H⋯Cl inter­actions lead to the formation of layers parallel to (002). C—H⋯π inter­actions also occur between these planes. Hirshfeld surface analyses were performed to investigate and qu­antify the inter­molecular inter­actions between the mol­ecules of all compounds.

## Abstract 2

Mol­ecules (**1**), (**2**), (**3**) and (**5**) adopt a *Z* configuration with respect to the central C=N bond, while (**4**) adopts an *E* configuration.

## 1. Chemical context

Catalytic olefination of hydrazones is a versatile method for the construction of halogenated alkenes starting from hydrazones (Adonin *et al.*, 2019 ▸; Bertani *et al.*, 2010 ▸; Metrangolo & Resnati, 2008 ▸; Askerova *et al.*, 2024 ▸, Sergeev *et al.*, 2020*a* ▸,*b* ▸). In the case of the reaction with N-substituted hydrazones the reaction leads to formation of di­chlorodi­aza­dienes (Nenajdenko *et al.*, 2017 ▸). By using carbon tetra­bromide for olefination it is possible to prepare di­bromo­substituted di­aza­dienes as well (Nenajdenko *et al.*, 2023 ▸). Recently, these type of building blocks attracted attention for preparation of numerous classes of nitro­gen-containing heterocycles with inter­esting properties (Vitaku *et al.*, 2014 ▸; Das *et al.*, 2019 ▸; Sergeev *et al.*, 2020*c* ▸; Tsyrenova *et al.*, 2023 ▸; Safronov *et al.*, 2023 ▸; Tsyrenova *et al.*, 2020*a* ▸,*b* ▸). It is particularly important to note that the solvolysis reaction of di­chlorodi­aza­dienes simultaneously yields *Z* and *E* isomers of aryl­hydrazones of α-keto esters (Shikhaliyev *et al.*, 2021*a* ▸).
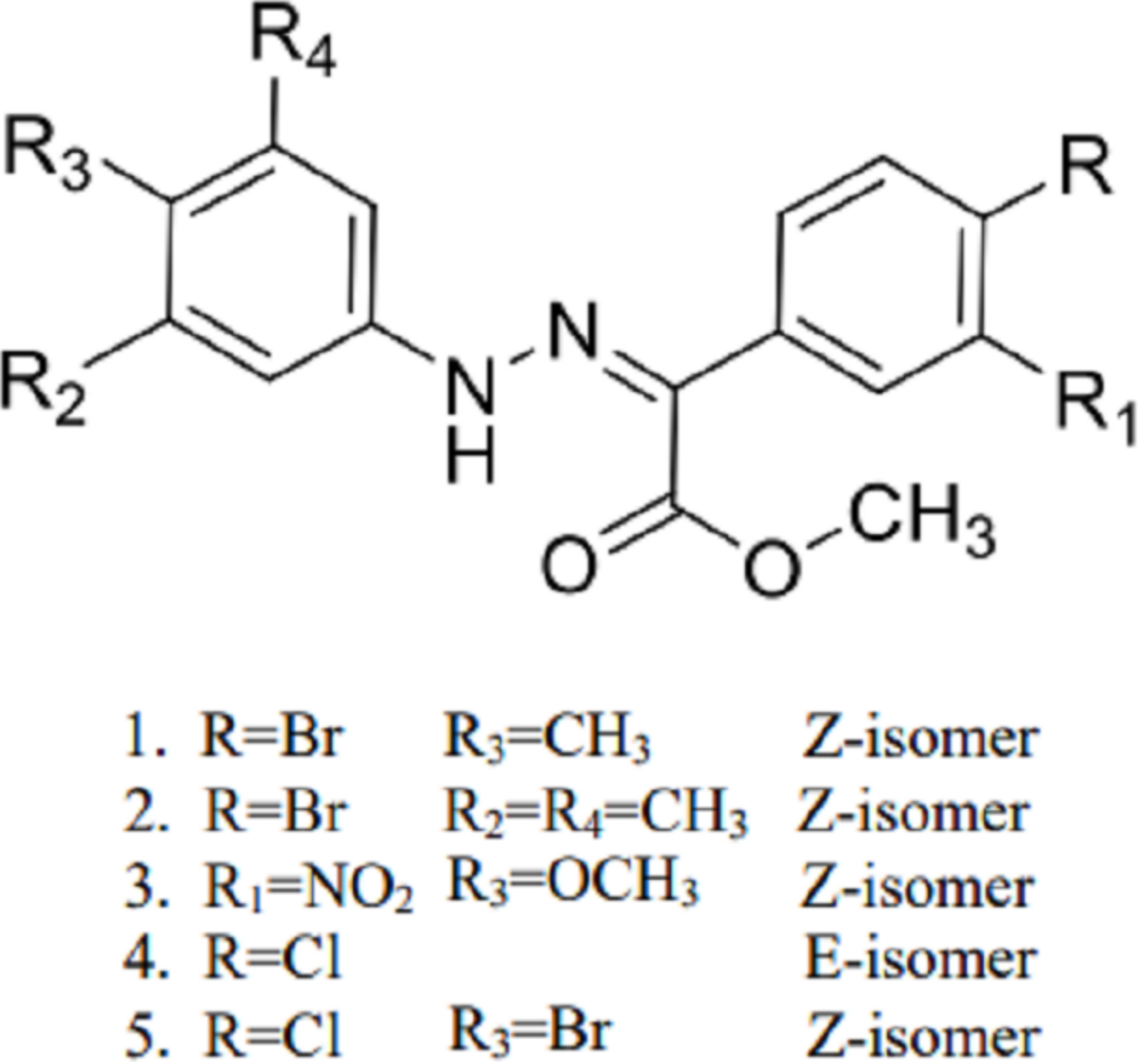


**Figure 1:**
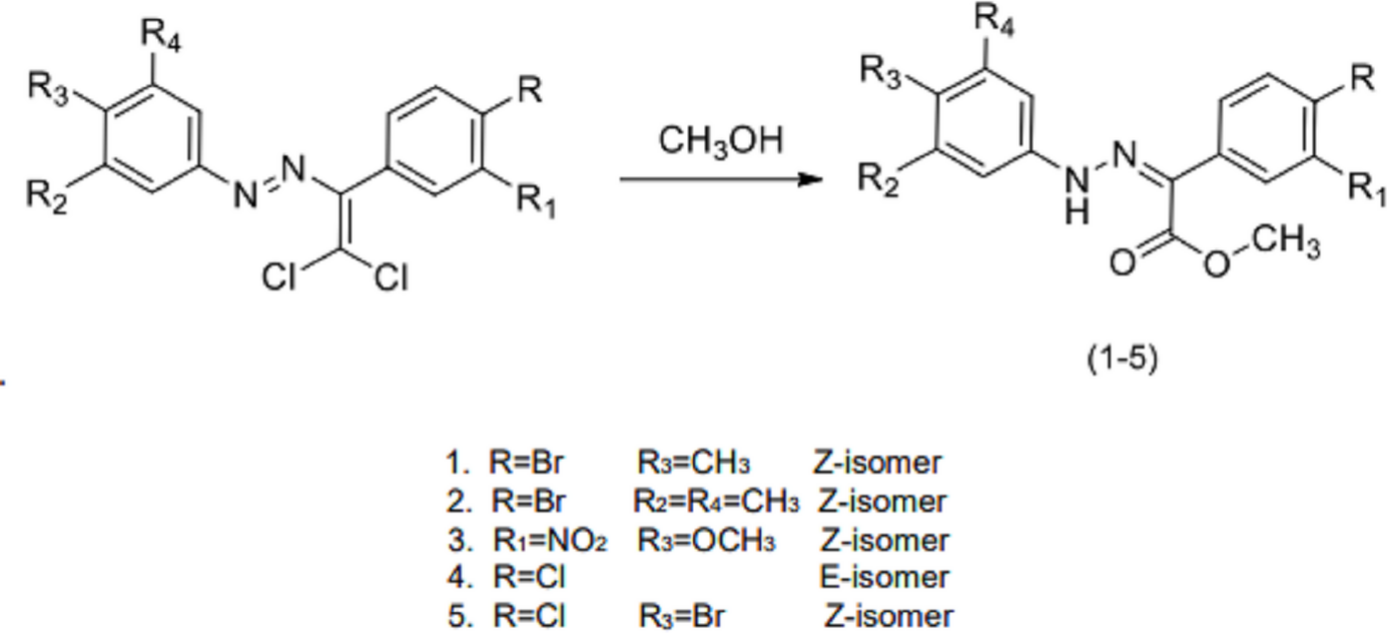
Schematic representation of the synthesis of compounds (**1**)–(**5**).

In this context, the methano­lysis reaction of some di­chlorodi­aza­dienes was carried out and the synthesis of aryl­hydrazo derivatives (**1**)–(**5**) of the corresponding α-keto esters was achieved (Fig. 1 ▸).

## 2. Structural commentary

**Figure 2:**
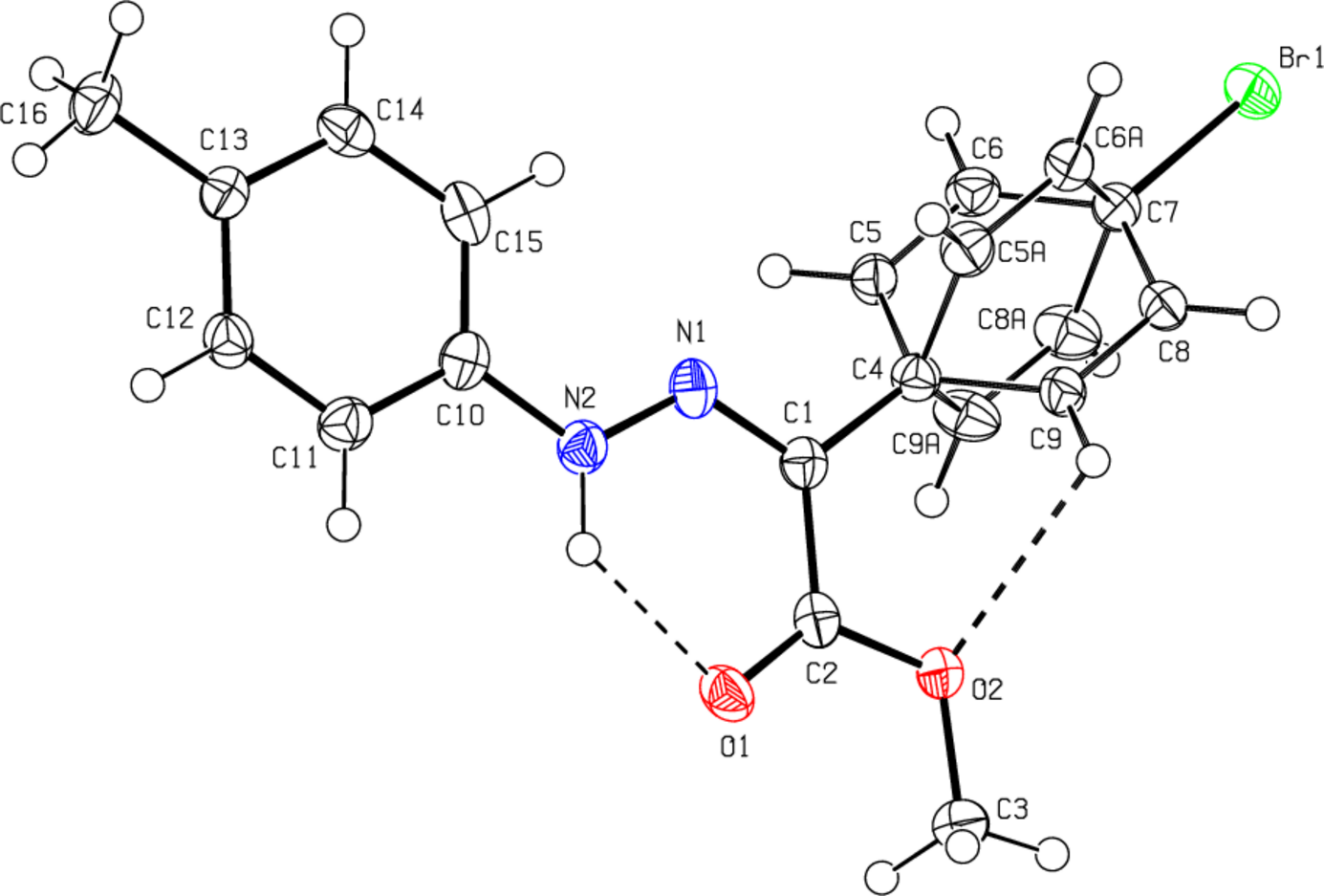
The mol­ecular structure of (**1**), showing the atom labelling and displacement ellipsoids drawn at the 50% probability level. The phenyl ring atoms of the bromo­phenyl group of (**1**) are disordered over two sets of sites with equal occupancies. N2—H2⋯O1 and C9—H9⋯O2 intra­molecular hydrogen bonds are shown by dashed lines.

**Figure 3:**
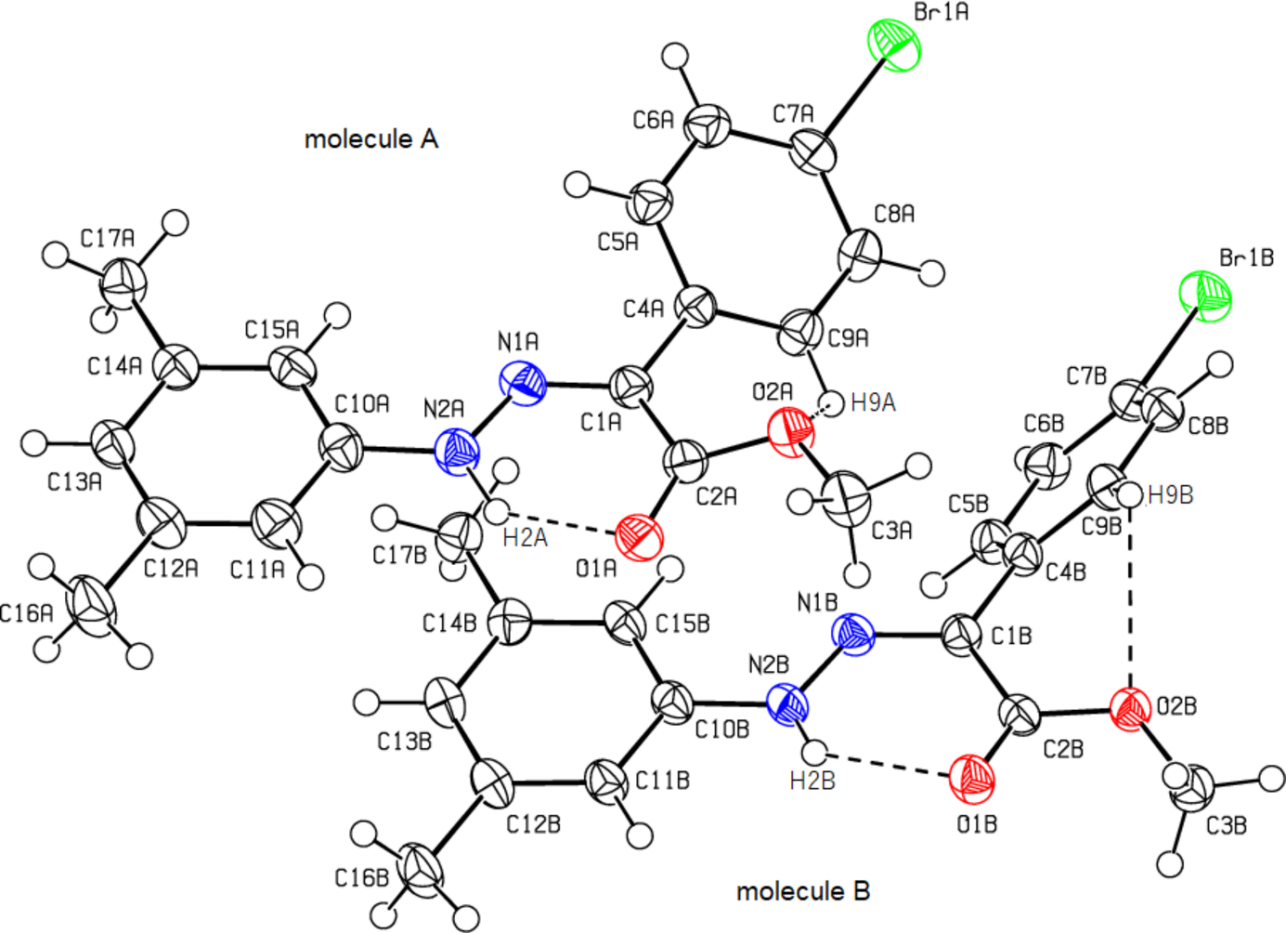
The two mol­ecules, *A* and *B*, in the asymmetric unit of (**2**), showing the atom labelling and displacement ellipsoids drawn at the 50% probability level.

**Figure 4:**
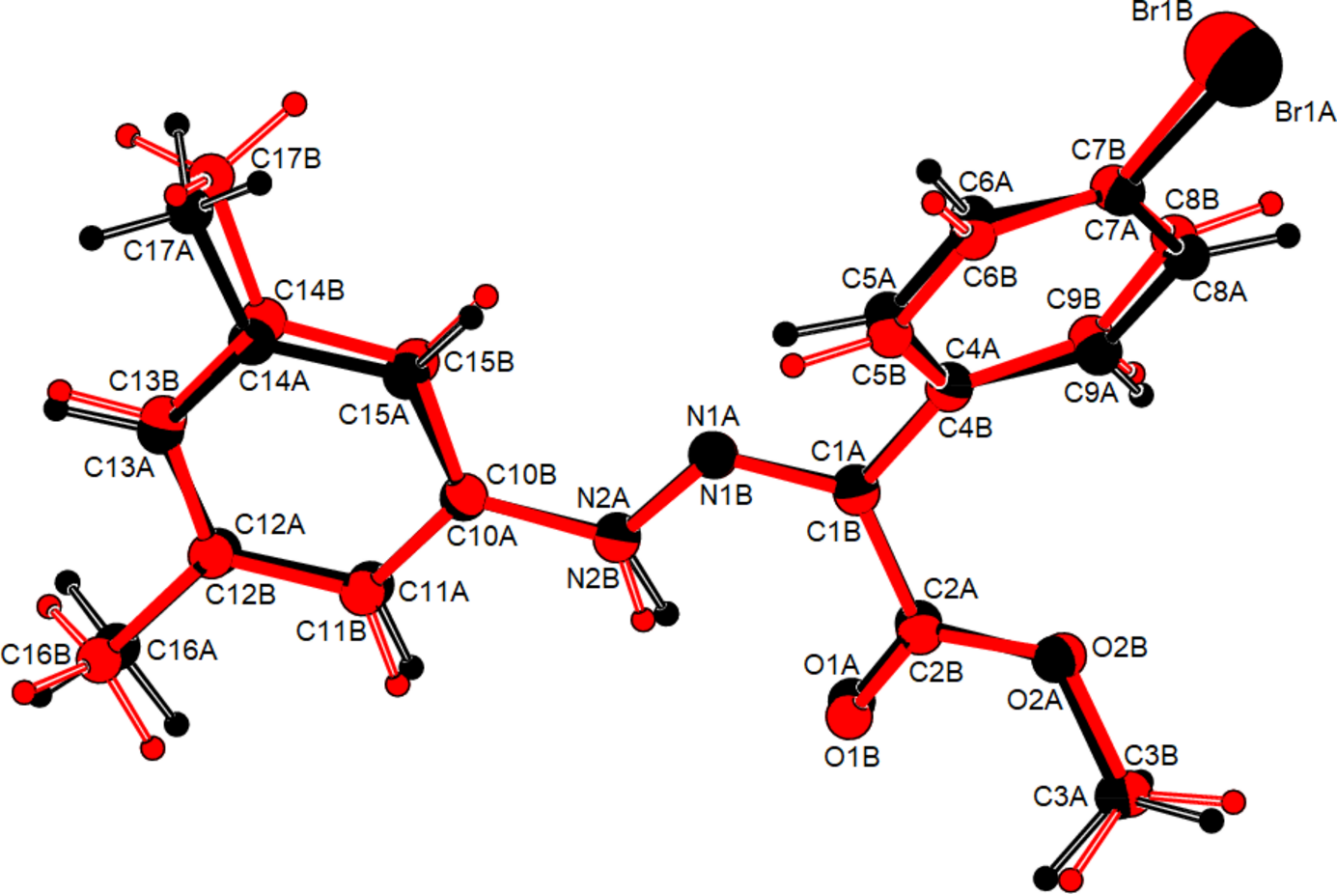
A least-squares overlay of the two independent mol­ecules *A* (black) and *B* (red) of (**2**).

**Figure 5:**
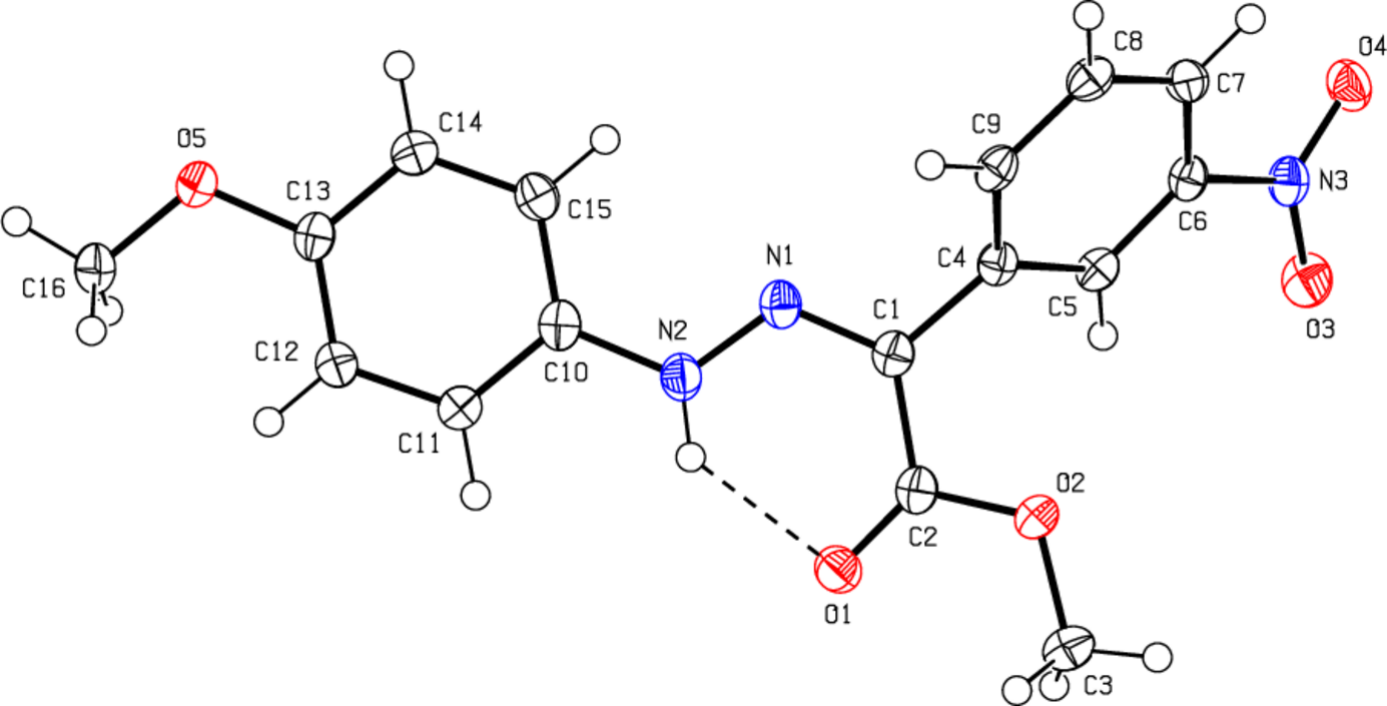
The mol­ecular structure of (**3**), showing the atom labelling and displacement ellipsoids drawn at the 50% probability level.

**Figure 6:**
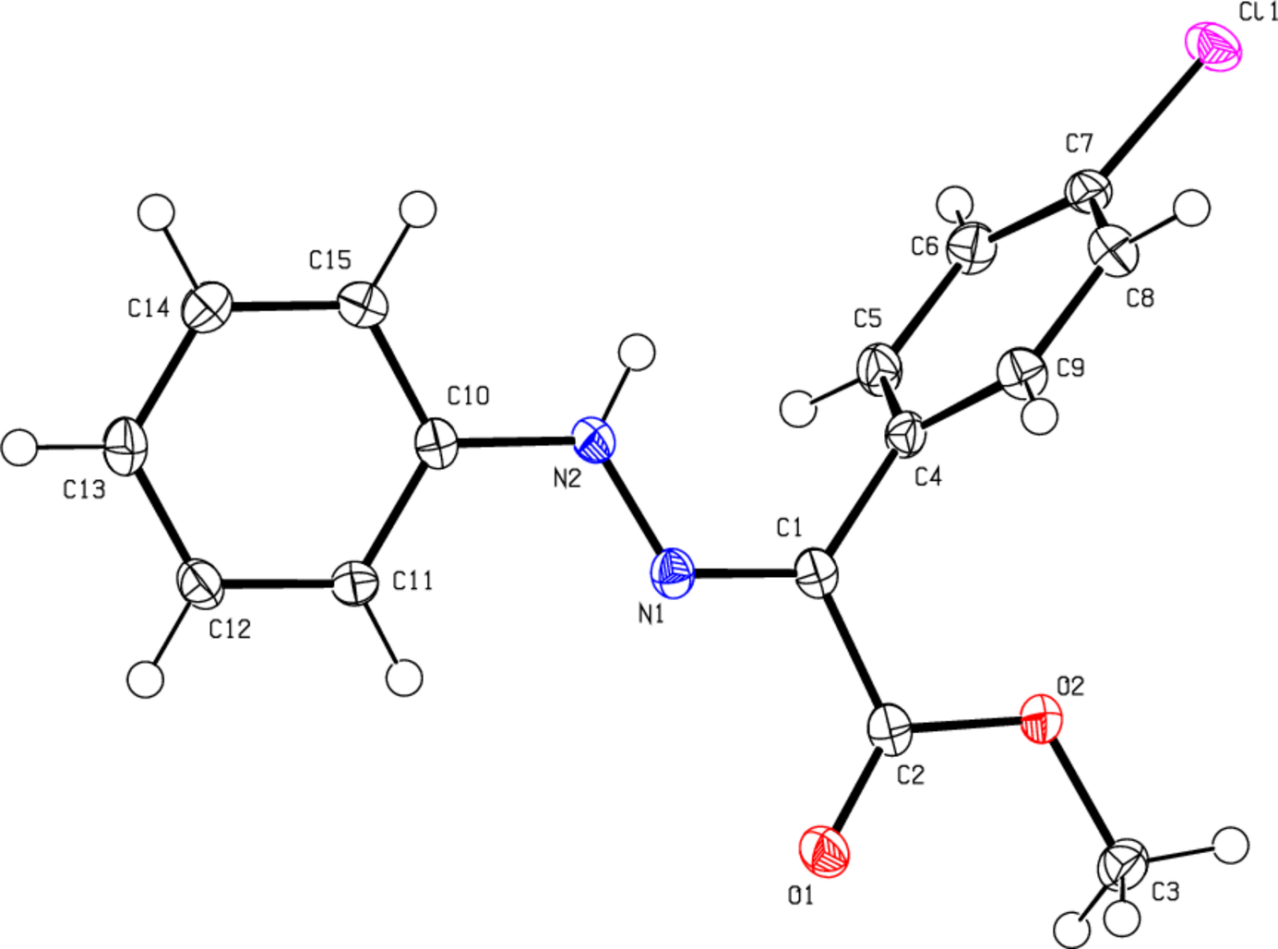
The mol­ecular structure of (**4**), showing the atom labelling and displacement ellipsoids drawn at the 50% probability level.

**Figure 7:**
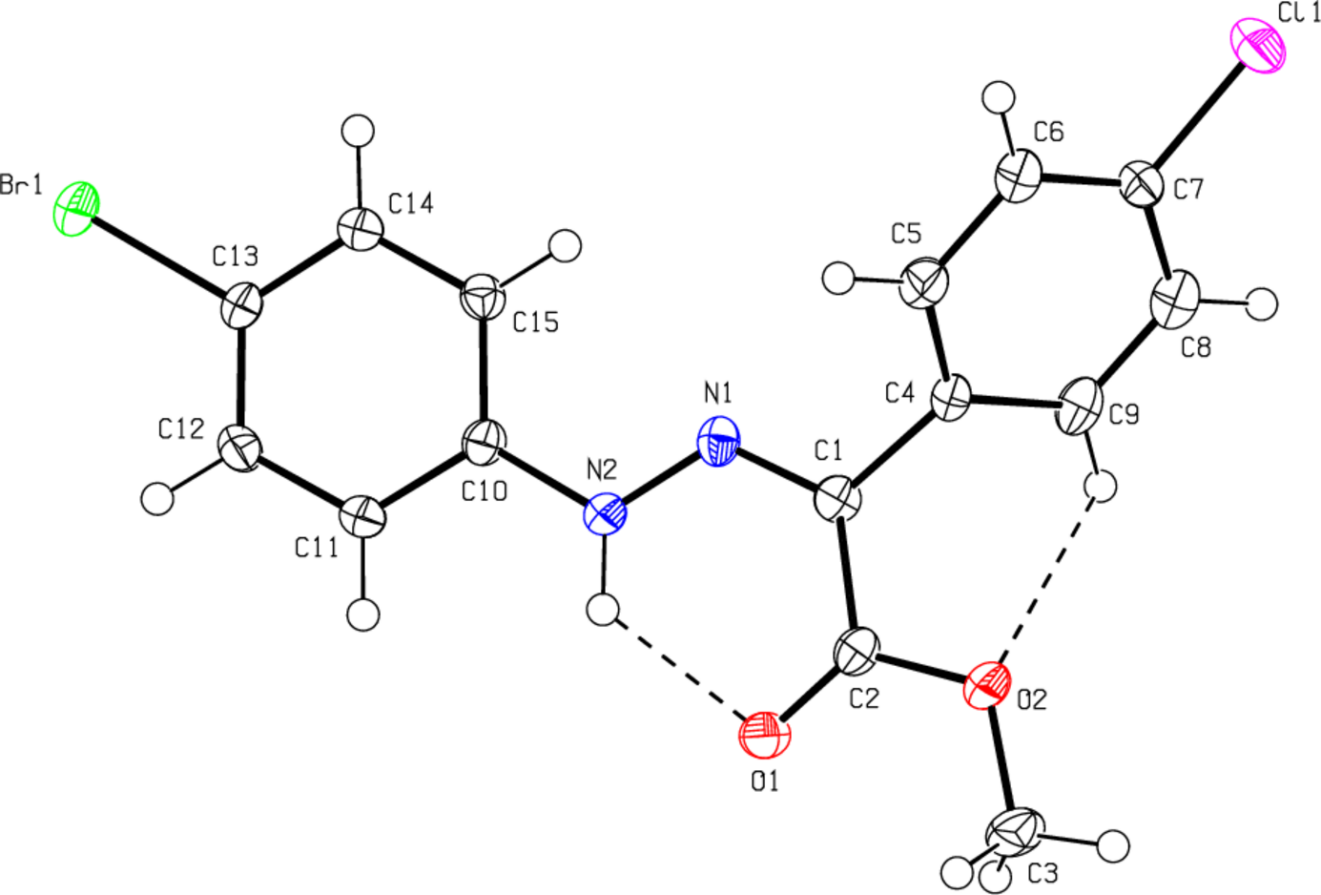
The mol­ecular structure of (**5**), showing the atom labelling and displacement ellipsoids drawn at the 50% probability level.

C_16_H_15_BrN_2_O_*2*_ (**1**) (Fig. 2 ▸) crystallizes in the monoclinic *C*2/*c* space group with *Z* = 8. The atoms of the phenyl ring of the bromo­phenyl group of (**1**) are disordered over two sets of sites with equal occupancies. C_17_H_17_BrN_2_O_2_ (**2**) (Fig. 3 ▸) crystallizes with two mol­ecules *A* and *B* in the asymmetric unit in the triclinic *P*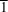 space group with *Z* = 4. An overlay fit of mol­ecule *B* on mol­ecule *A* of (**2**) is shown in Fig. 4 ▸; the weighted r.m.s. fit of the 22 non-H atoms is 0.268 Å with the major differences in the phenyl groups (C4*A*–C9*A*/C4*B*–C9*B* and C10*A*–C15*A*/C10*B*–C15*B*) of mol­ecules *A* and *B*. C_16_H_15_N_3_O_5_ (**3**) (Fig. 5 ▸) crystallizes in the monoclinic *C*2/*c* space group with *Z* = 8, C_15_H_13_ClN_2_O_2_ (**4**) (Fig. 6 ▸) crystallizes in the ortho­rhom­bic *Pbca* space group with *Z* = 8, and C_15_H_12_BrClN_2_O_2_ (**5**) (Fig. 7 ▸) crystallizes in the ortho­rhom­bic *Pca*2_1_ space group with *Z* = 4.

Mol­ecules (**1**), (**2**), (**3**) and (**5**) adopt a *Z* configuration with respect to the central C=N bond, while (**4**) adopts an *E* configuration. This also affects intra- and inter­molecular hydrogen-bonding and, consequently, the packing arrangement (see next section for details). The mol­ecular shapes of compounds (**1**), (**2**) and (**5**) are stabilized by intra­molecular N—H⋯O and C—H⋯O hydrogen bonds (Tables 1 ▸, 2 ▸ ▸ ▸, 5 ▸), forming *S*(6) ring motifs (Bernstein *et al.*, 1995 ▸), while the stability of mol­ecule (**3**) is provided only by intra­molecular N—H⋯O inter­actions (Table 3 ▸) with the same kind of hydrogen-bonding pattern. In the mol­ecule of (**4**) intra­molecular hydrogen bonds do not occur. In the five mol­ecules, the angles between the phenyl rings connected by the —NH—N=C— bridge are different. The corresponding angle is 44.40 (18)° for (**1**) for one of the two orientations in the disordered parts and 52.74 (19)° for the other orientation, while the dihedral angle between the disordered phenyl rings in (**1**) is 83.1 (2)°. In (**2**), the angle is 32.31 (18)° for mol­ecule *A* and 45.62 (18)° for mol­ecule *B*. In (**3**) it is 51.09 (7)°, in (**4**) 83.69 (6)° and in (**5**) 49.9 (3)°. Other bond lengths and angles within the five mol­ecules are in normal ranges and consistent with those of the related compounds described in the *Database survey* (Section 4).

## 3. Supra­molecular features and Hirshfeld surface analysis

**Figure 8:**
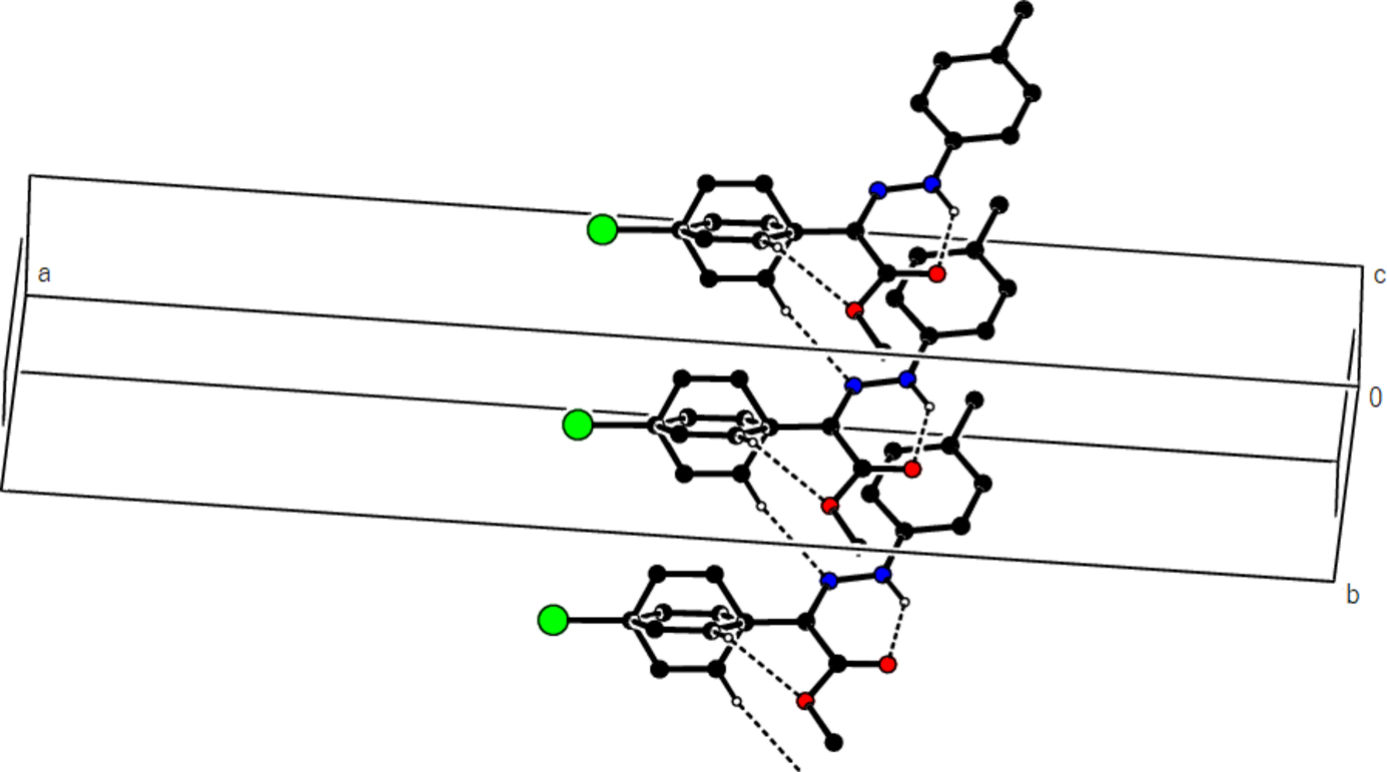
View of the intra- and inter­molecular hydrogen bonds of (**1**) along the *b* axis.

**Figure 9:**
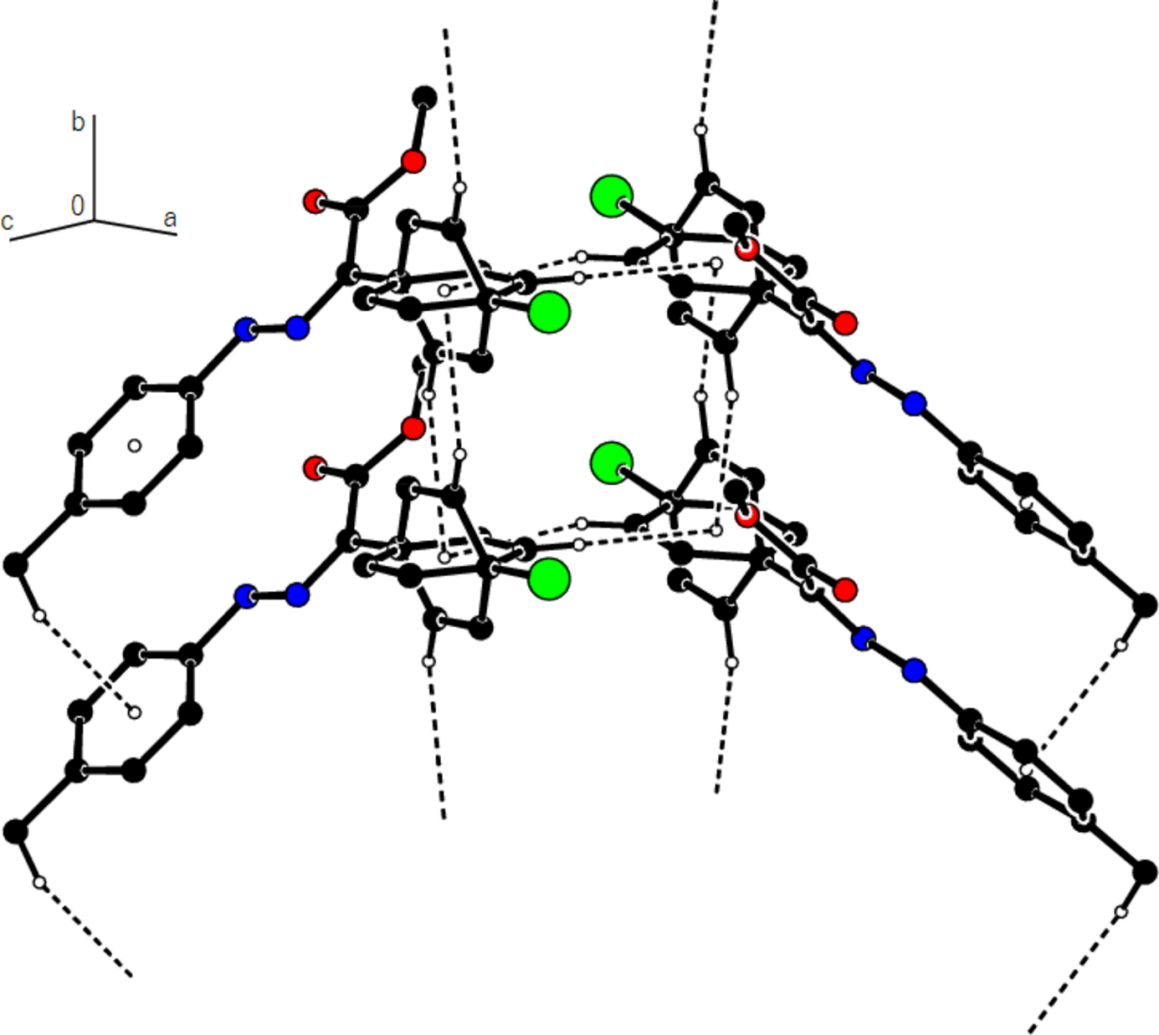
View of the C—H⋯π inter­actions of (**1**) in the unit cell along the *b* axis.

**Figure 10:**
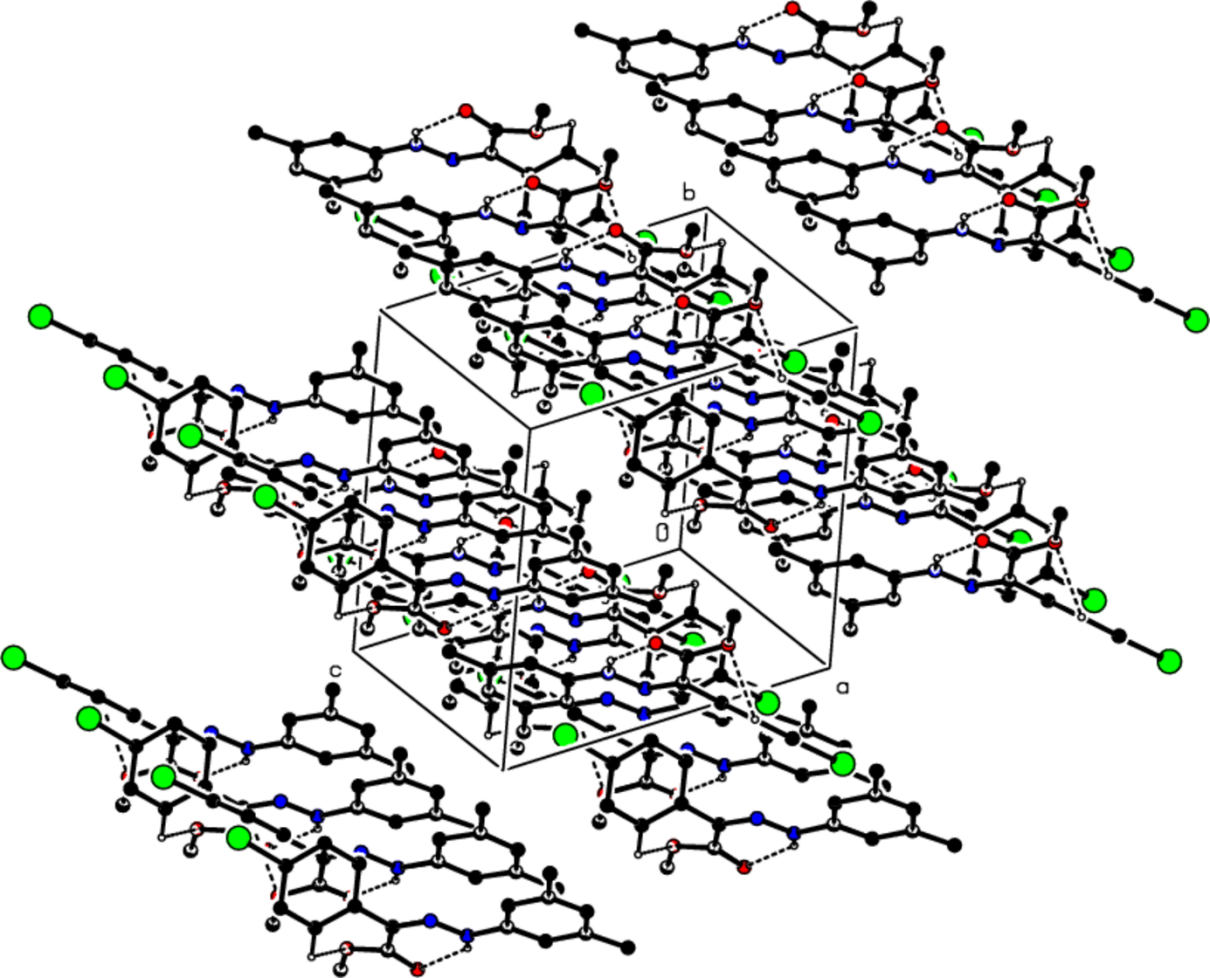
A general view of the mol­ecular packing of (**2**) in the unit cell.

**Figure 11:**
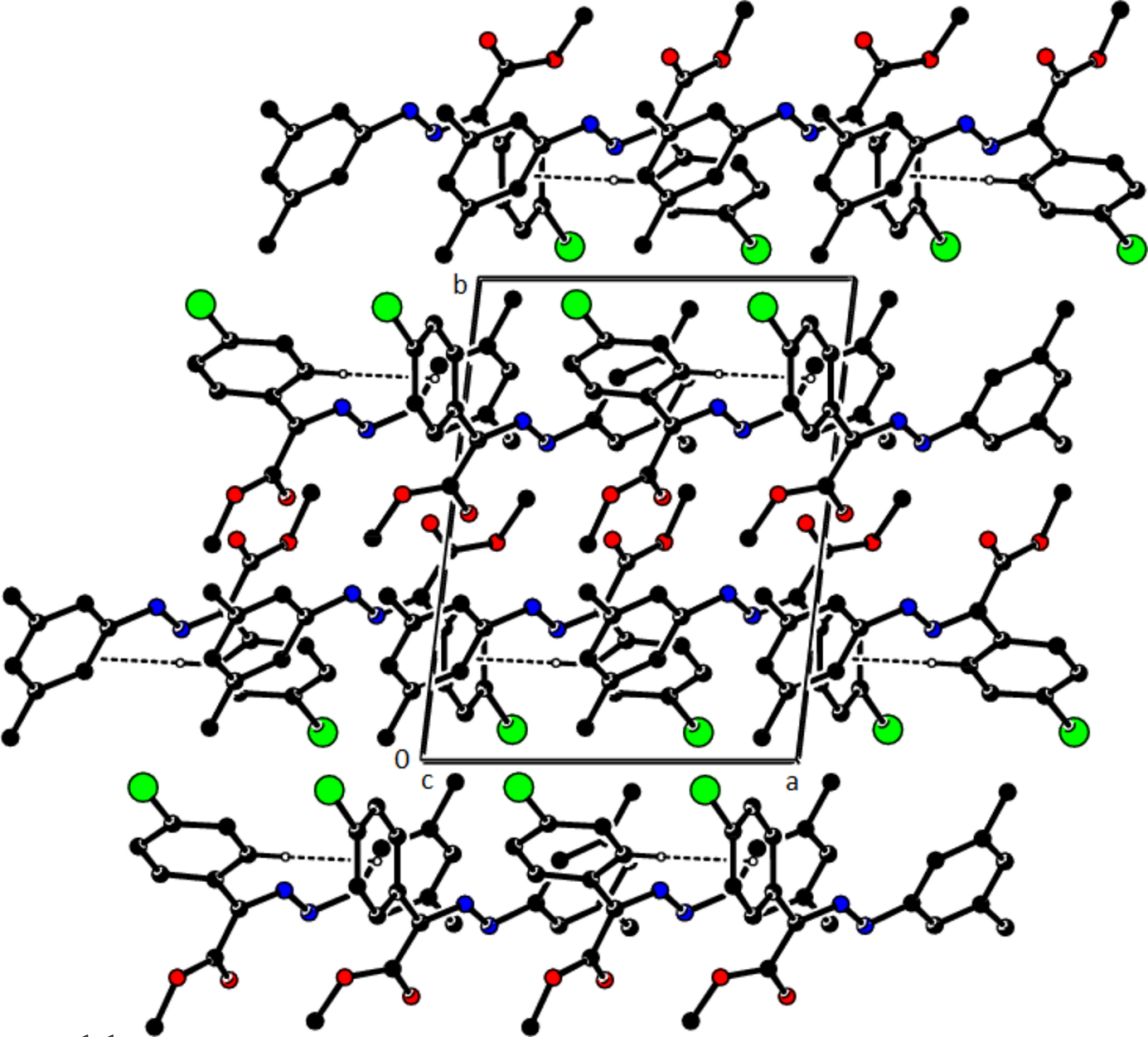
A view of the C—H⋯π inter­actions of (**2**) along the *c* axis.

**Figure 12:**
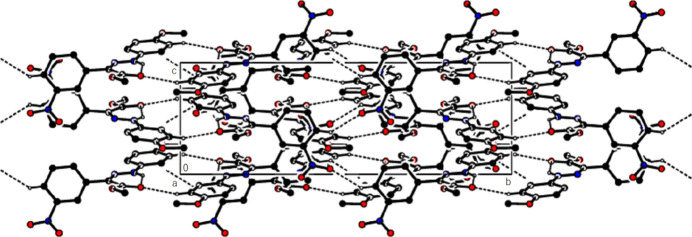
A view of the packing of (**3**) along the *a* axis.

**Figure 14:**
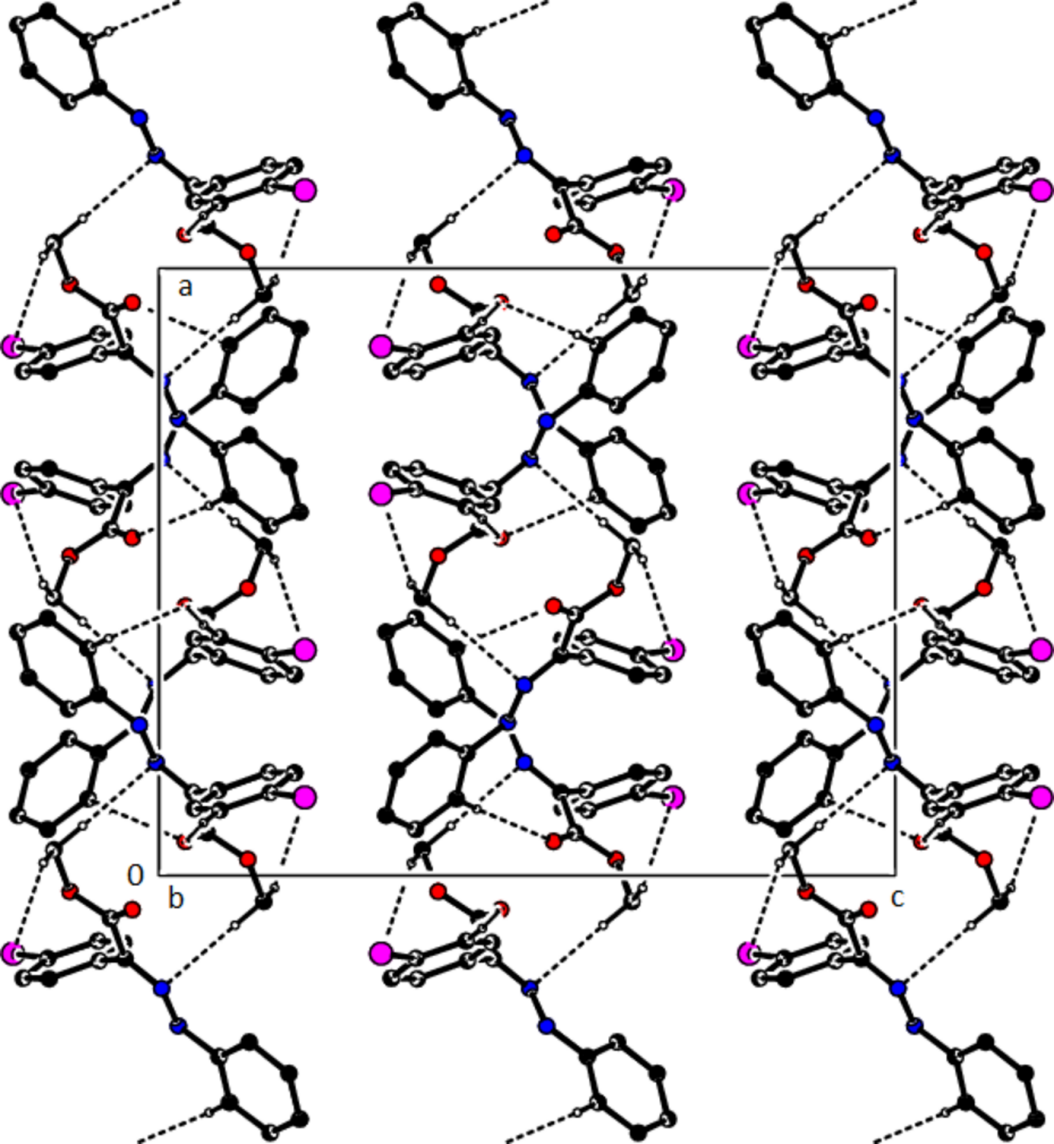
A view of the hydrogen bonds present in (**4**) in a view along the *b* axis.

**Figure 16:**
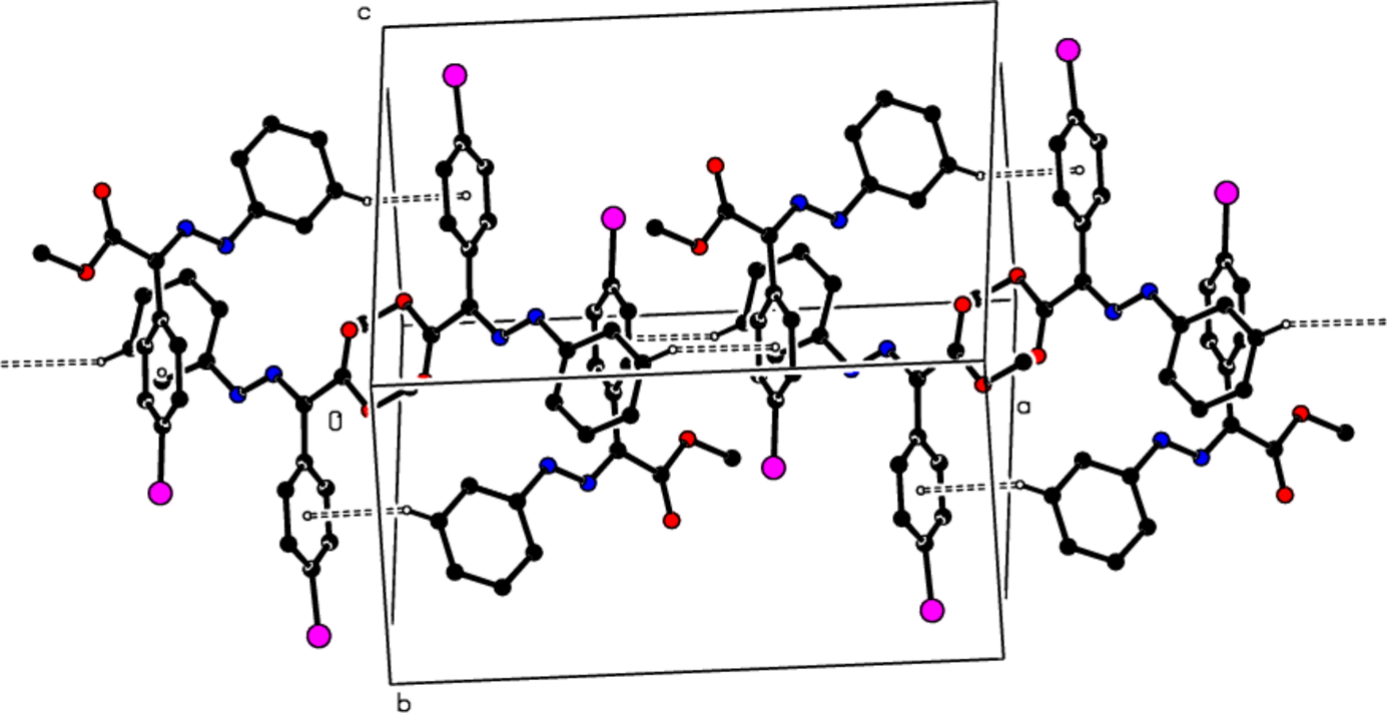
A view of the C—H⋯π contacts of (**4**) along the *a* axis.

**Figure 17:**
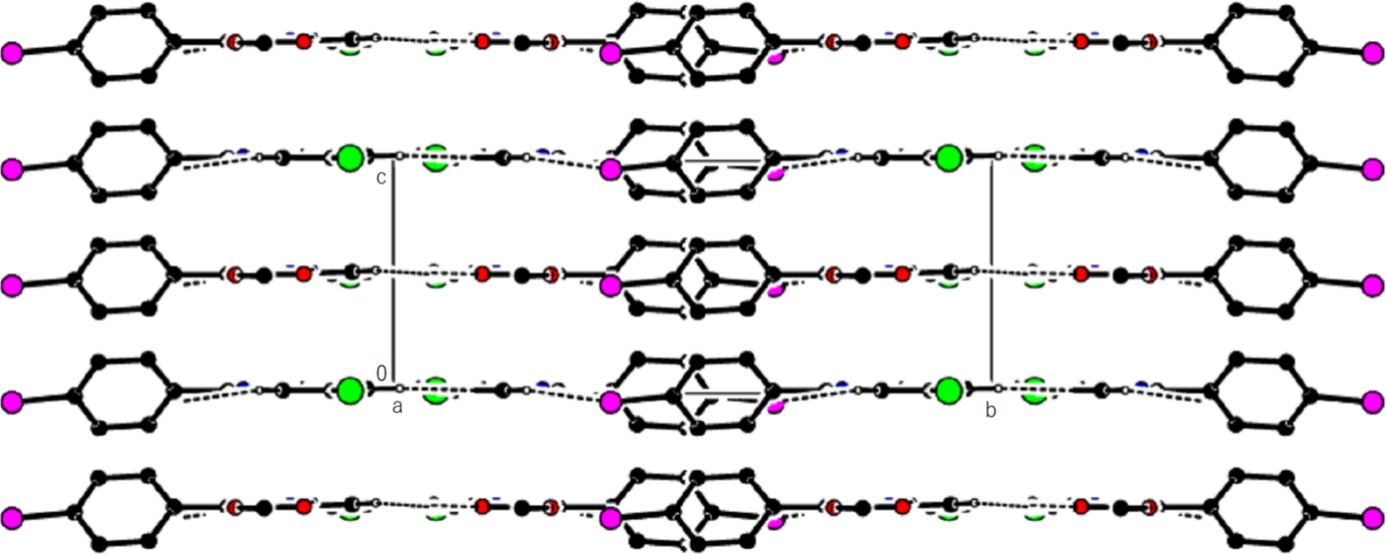
A view of the hydrogen bonds present in (**5**) in a view along the *a* axis.

**Figure 19:**
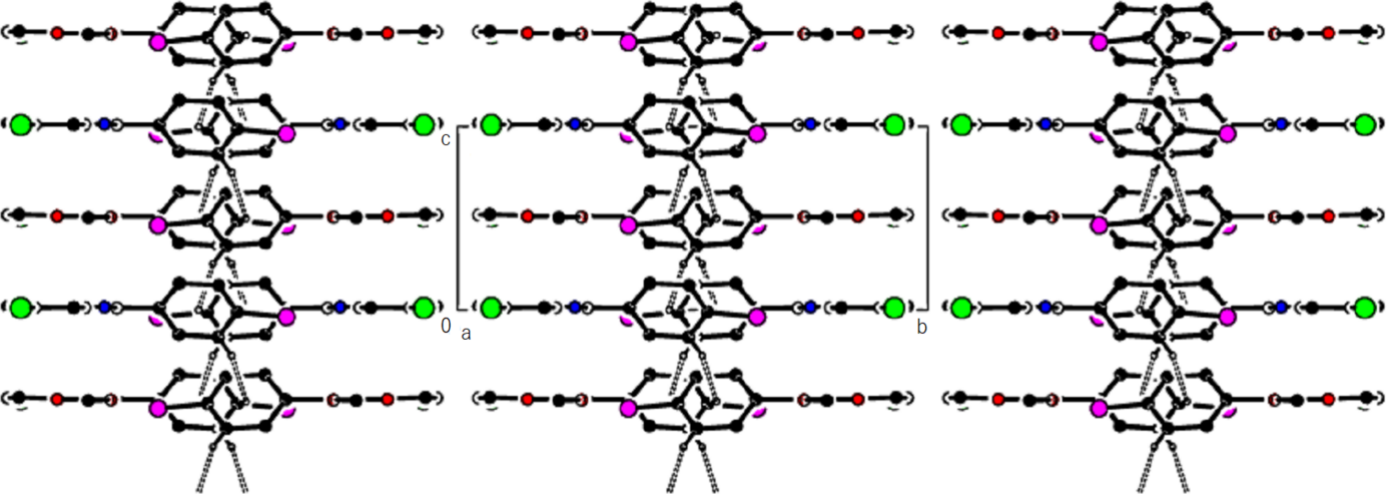
A view of the C—H⋯π contacts of (**5**) in a view along the *a* axis.

In the crystal of (**1**), non-classical C9*A*—H9*A*⋯N1 hydrogen bonds connect adjacent mol­ecules parallel to [010] to form *C*(5) chains (Table 1 ▸; Fig. 8 ▸). In addition, mol­ecules are connected by C—H⋯π inter­actions to form ribbons along the propagation direction (Fig. 9 ▸). Significant inter­molecular hydrogen bonding is not observed in (**2**). The mol­ecules are aligned in ribbons parallel to [100] in the (010) plane (Fig. 10 ▸) whereby pairs of mol­ecules are formed by C5—H5⋯*Cg*3 inter­actions (Table 2 ▸; Fig. 11 ▸). The crystal structure is consolidated through van der Waals inter­actions. In the crystal of (**3**), C7—H7⋯O4 and C12—H12⋯O1 inter­actions form ribbons along [010] (Table 3 ▸; Figs. 12 ▸ and 13 ▸) , but C—H⋯π inter­actions are not observed. The crystal structure is consolidated through van der Waals inter­actions between the ribbons. In the crystal structure of (**4**), C3—H3*B*⋯N1, C3—H3*C*⋯Cl1, C6⋯H6⋯O1 and C15—H15⋯O1 inter­molecular inter­actions connect the mol­ecules under formation of layers parallel to the (001) plane (Table 4 ▸; Figs. 14 ▸, 15 ▸). At the same time, C14—H14⋯*Cg*1 inter­actions link the mol­ecules together in the (001) plane along [100] (Fig. 16 ▸). The crystal structure is consolidated by van der Waals inter­actions between the layers. In the crystal structure of (**5**), C11—H11⋯Br1, C12—H12⋯O1 and C14—H14⋯Cl1 inter­molecular hydrogen bonds form layers parallel to (002) (Table 5 ▸; Figs. 17 ▸, 18 ▸). C8—H8⋯*Cg*1 inter­actions also take place between these planes and consolidate the crystal structure (Fig. 19 ▸).

**Figure 20:**
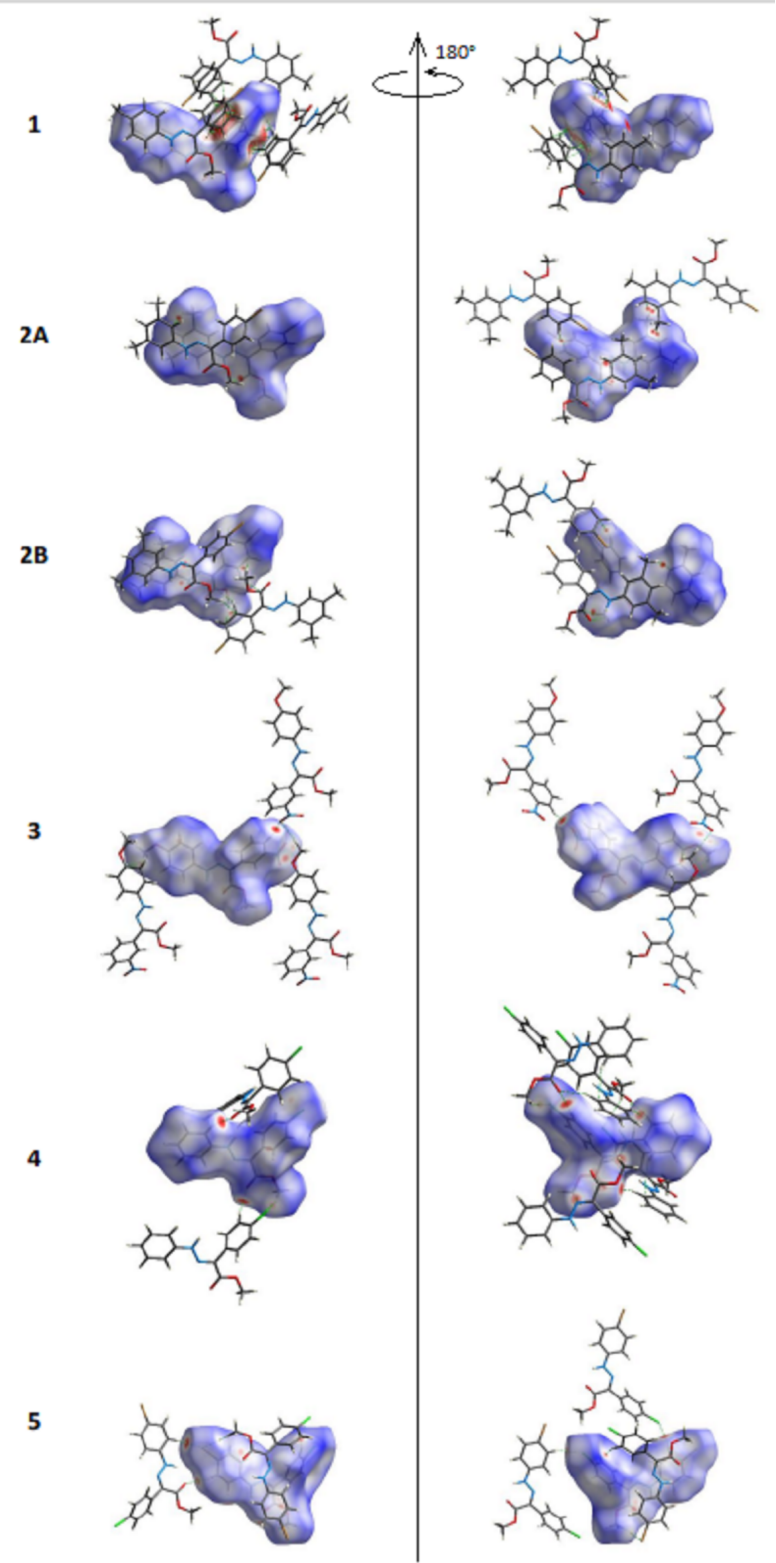
Front (*a*) and back (*b*) views of the three-dimensional Hirshfeld surface of the mol­ecules (**1**), (**2**), (**3**), (**4**) and (**5**), with some C—H⋯O, C—H⋯Br, C—H⋯Cl and O—H⋯O hydrogen bonds shown as dashed lines.

**Figure 21:**
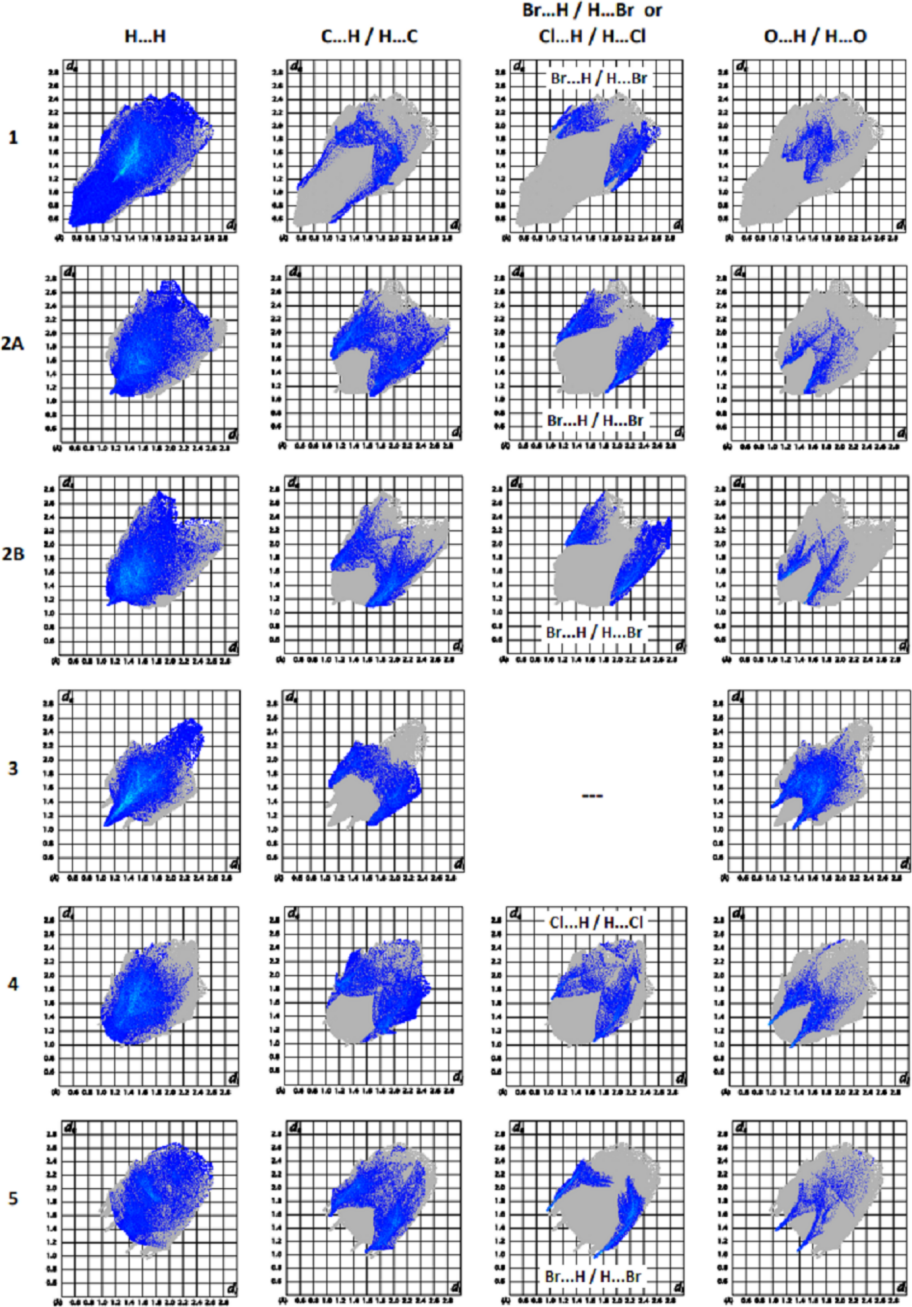
The full two-dimensional fingerprint plots for (**1**), (**2**), (**3**), (**4**) and (**5**), showing (*a*) H⋯H, (*b*) C⋯H/H⋯C, (*c*) Cl⋯H/H⋯Cl or Br⋯H/H⋯Br and (*d*) O⋯H/H⋯O inter­actions. The *d*_i_ and *d*_e_ values are the closest inter­nal and external distances (in Å) from given points on the Hirshfeld surface.

To qu­antify the inter­molecular inter­actions between the mol­ecules in (**1**)–(**5**) in their respective crystal structures, Hirshfeld surfaces (Fig. 20 ▸) and their corresponding two-dimensional fingerprint plots (Fig. 21 ▸) were calculated with *CrystalExplorer* (Spackman *et al.*, 2021 ▸). The dominant inter­actions in all compounds are H⋯H [(**1**): 59.9%, (**2***A*): 41.8%, (**2***B*): 46.4, (**3**): 38.9%, (**4**): 39.0% and (**5**): 26.3%] and C⋯H/H⋯C [(**1**): 13.3%, (**2***A*): 26.8%, (**2***B*): 21.0, (**3**): 16.0%, (**4**): 21.4% and (**5**): 25.1%]. In (**3**) and (**4**), O⋯H/H⋯O inter­actions are also important inter­actions [(**3**): 28.5% and (**4**): 12.7%]. Br⋯H/H⋯Br in Br-containing compounds (**1**), (**2**) and (**5**) [(**1**): 12.5%, (**2***A*): 15.7%, (**2***B*): 15.6% and (**5**): 15.8%] and Cl⋯H/H⋯Cl inter­actions in Cl-containing compound (**5**) [(**5**): 14.5%] also contribute to the stability of the crystal structures. The full percentage contributions of inter­atomic contacts calculated for each compound are given in Table 6 ▸. The presence of different functional groups in the compounds leads to some differences in the remaining weak inter­actions.

## 4. Database survey

A search of the Cambridge Structural Database (CSD, Version 5.42, update of September 2021; Groom *et al.*, 2016 ▸) for structures with the (1E)-1-benzyl­idene-2-phenyl­hydrazine moiety revealed that the three most similar compounds are KOGYEN (Akhramez *et al.*, 2019 ▸), UREKIM (Jasinski *et al.*, 2011 ▸) and SOJQAL (Sultan *et al.*, 2014 ▸).

KOGYEN crystallizes in the monoclinic *Cc* space group with *Z* = 4, UREKIM in the triclinic *P*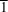 space group with *Z* = 2, and SOJQAL in the ortho­rhom­bic *P*2_1_2_1_2_1_ space group with *Z* = 4.

In KOGYEN, mol­ecules are linked by a C—H⋯π-phenyl inter­action, forming zigzag chains propagating along [100]. The N—H group does not participate in hydrogen bonding but is directed towards the phenyl ring of an adjacent mol­ecule, so linking the chains *via* weak N—H⋯π inter­actions into the three-periodic structure. In UREKIM, crystal packing is stabilized by N—H⋯O hydrogen bonds, weak C—H⋯O and C—H⋯F inter­molecular inter­actions and centroid-to-centroid π-ring stacking inter­actions. In SOJQAL, mol­ecules are linked by N—H⋯O and C—H⋯O hydrogen bonds into zigzag chains propagating along [100].

## 5. Synthesis and crystallization

Compounds (**1**), (**2**) (**3**), (**4**) and (**5**) were synthesized according to a literature protocol (Shikhaliyev *et al.*, 2021*b* ▸). For the procedure, 10 mg of the corresponding di­chlorodi­aza­diene and 30 ml of methanol were mixed and stirred for 2 h. The residue was purified by column chromatography on silica gel using appropriate mixtures of hexane and di­chloro­methane (1/1 *v*/*v*), and corresponding ethers were obtained as polycrystalline yellow solids.

**Methyl (2*****Z*****)-(4-bromo­phen­yl)[2-(4-methyl­phen­yl)hydrazinyl­idene]acetate** (**1**): yield 75%; m.p. 370 K. ^1^H NMR (300 MHz, chloro­form-*d*, ppm) δ 12.48 (*s*, 1H, –NH), 7.53 (*d*, *J* = 2.8 Hz, 4H, Ar), 7.19 (*t*, *J* = 7.0 Hz, 4H, Ar), 3.89 (*s*, 3H, –OCH_3_), 2.34 (*s*, 3H, -*-*CH_3_). ^13^C NMR (75 MHz, CDCl_3_, ppm) δ 140.8, 140.6, 135.4, 132.5, 131.0, 130.1, 129.9, 121.5, 114.3, 114.1, 51.7, 20.8

**Methyl (2*****Z*****)-(4-bromo-phen­yl)[2-(3,5-di­methyl­phen­yl)hydrazinyl­idene]acetate** (**2**): yield 37%; m.p. 383 K. ^1^H NMR (300 MHz, chloro­form-*d*, ppm) δ 12.44 (*s*, 1H, –NH), 7.54 (*s*, 4H, Ar), 6.93 (*s*, 2H, Ar), 6.71 (*s*, 1H, Ar), 3.89 (*s*, 3H, –OCH_3_) , 2.34 (*s*, 6H, –CH_3_,). ^13^C NMR (75 MHz, chloro­form-*d*, ppm) δ 163.8, 142.8, 139.2, 135.4, 131.0, 130.2, 126.0, 124.8, 121.6, 112.2, 51.8, 21.4.

**Methyl (2*****Z*****)-[2-(4-meth­oxy­phen­yl)hydrazinyl­idene](3-nitro­phen­yl)acetate** (**3**): yield 63%; m.p. 375.18 K. ^1^H NMR (300 MHz, chloro­form-*d*, ppm) δ 12.67 (*s*, 1H, –NH), 8.55 (*s*, 1H, Ar), 8.14 (*dd*, *J* = 8.2, 1.3 Hz, 1H, Ar), 8.01 (*d*, *J* = 7.9 Hz, 1H, Ar), 7.53 (*t*, *J* = 8.0 Hz, 1H, Ar), 7.27 (*d*, *J* = 2.1 Hz, 1H, Ar), 7.25 (*d*, *J* = 2.0 Hz, 1H, Ar), 6.96–6.89 (*m*, 2H, Ar), 3.92 (*s*, 3H, –OCH_3_), 3.82 (*s*, 3H, –OCH_3_). ^13^C NMR (75 MHz, CDCl_3_, ppm) δ 163.7, 156.2, 148.0, 138.2, 136.3, 134.1, 128.6, 123.6, 123.2, 121.7, 115.8, 114.8, 55.6, 51.9.

**Methyl (2*****E*****)-(4-chloro-phen­yl)(2-phenyl­hydrazinyl­idene)acetate** (**4**): yield 63%; m.p. 375 K. ^1^H NMR (300 MHz, chloro­form-*d*, ppm) δ 8.07 (*s*, 1H, –NH), 7.55 (*d*, *J* = 8.4 Hz, 2H, Ar), 7.30 (*dd*, *J* = 7.8, 5.4 Hz, 4H, Ar), 7.16 (*d*, *J* = 7.7 Hz, 2H, Ar), 7.01 (*t*, *J* = 7.3 Hz, 1H, Ar), 3.88 (*s*, 3H, –OCH_3_). ^13^C NMR (75 MHz, CDCl_3_) δ 163.8, 142.9, 134.8, 133.5, 129.8, 129.4, 128.0, 126.4, 122.8, 114.3, 77.5, 77.0, 76.7, 76.6, 51.8.

**Methyl (2*****Z*****)-[2-(4-bromo­phen­yl)-hydrazinyl­idene](4-chloro­phen­yl)acetate** (**5**): yield 42%; m.p. 382 K. ^1^H NMR (300 MHz, chloro­form-*d*, ppm) δ 12.43 (*s*, 1H, –NH), 7.58 (*d*, *J* = 8.3 Hz, 2H, Ar), 7.44 (*d*, *J* = 8.4 Hz, 2H, Ar), 7.37 (*d*, *J* = 8.2 Hz, 2H, Ar), 7.16 (*d*, *J* = 8.4 Hz, 2H, Ar), 3.90 (*s*, 3H, –OCH_3_). ^13^C NMR (75 MHz, CDCl_3_) δ 132.34, 132.29, 129.91, 129.88, 128.20, 128.15, 117.52, 115.92, 63.76, 52.02, 29.72.

Compounds (**1**), (**2**) (**3**), (**4**) and (**5**) were dissolved in di­chloro­methane and then left at room temperature for slow evaporation; red single crystals of all compounds suitable for X-ray diffraction analysis started to form after *ca* 2 d.

## 6. Refinement

Crystal data, data collection and structure refinement details are summarized in Table 7 ▸. The Moscow synchrotron radiation source was used to collect the data for crystals (**2**) and (**5**), while the data for crystals (**1**), (**3**) and (**4**) were collected using Cu *K*α radiation on a laboratory diffractometer. In all five compounds, C-bound H atoms were positioned geometrically and treated as riding atoms, with C—H = 0.95 and 0.98 Å and *U*_iso_(H) = 1.2*U*_eq_(C) or 1.5*U*_eq_(C-meth­yl). The NH group hydrogen atoms were found by difference-Fourier maps for all five crystals and were refined freely for (**1**), (**4**) and (**5**), while those in (**2**) and (**3**) were refined with *U*_iso_(H) = 1.2*U*_eq_(N) of the attached nitro­gen atom. In (**1**), the phenyl ring atoms of the bromo­phenyl group are disordered over two sets of sites with equal occupancies. In (**2**) owing to poor agreement between observed and calculated intensities, 23 reflections were omitted from the final cycles of refinement.

## Supplementary Material

Crystal structure: contains datablock(s) 1, 2, 3, 4, 5, global. DOI: 10.1107/S2056989025002051/wm5749sup1.cif

Structure factors: contains datablock(s) 1. DOI: 10.1107/S2056989025002051/wm57491sup7.hkl

Structure factors: contains datablock(s) 2. DOI: 10.1107/S2056989025002051/wm57492sup8.hkl

Structure factors: contains datablock(s) 3. DOI: 10.1107/S2056989025002051/wm57493sup9.hkl

Structure factors: contains datablock(s) 4. DOI: 10.1107/S2056989025002051/wm57494sup10.hkl

Structure factors: contains datablock(s) 5. DOI: 10.1107/S2056989025002051/wm57495sup11.hkl

Supporting information file. DOI: 10.1107/S2056989025002051/wm57491sup7.cml

Supporting information file. DOI: 10.1107/S2056989025002051/wm57492sup8.cml

Supporting information file. DOI: 10.1107/S2056989025002051/wm57493sup9.cml

Supporting information file. DOI: 10.1107/S2056989025002051/wm57494sup10.cml

Supporting information file. DOI: 10.1107/S2056989025002051/wm57495sup11.cml

CCDC references: 2428835, 2428834, 2428833, 2428832, 2428831

Additional supporting information:  crystallographic information; 3D view; checkCIF report

## Figures and Tables

**Figure 13:**
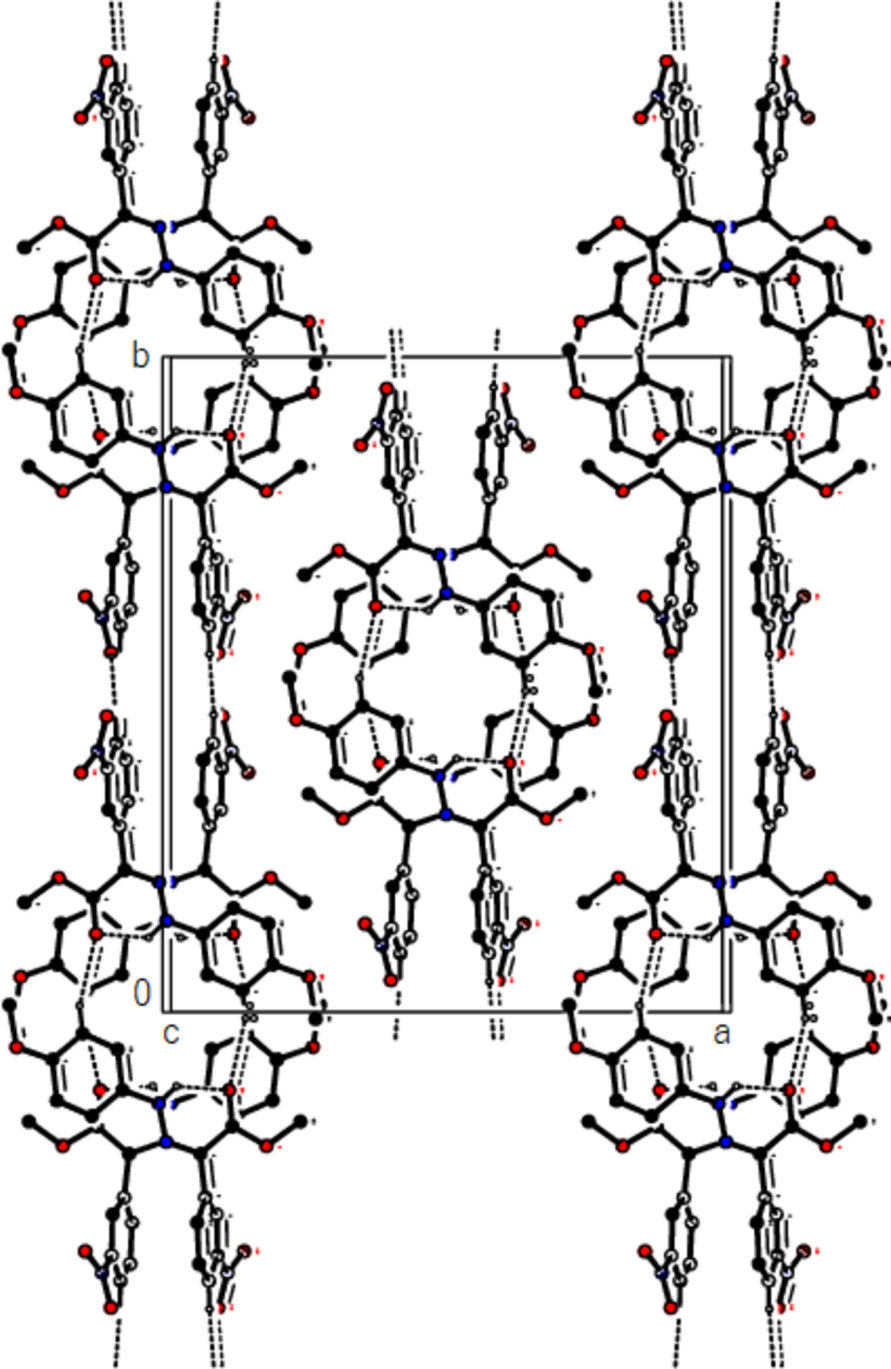
A view of the packing of (**3**) along the *c* axis.

**Figure 15:**
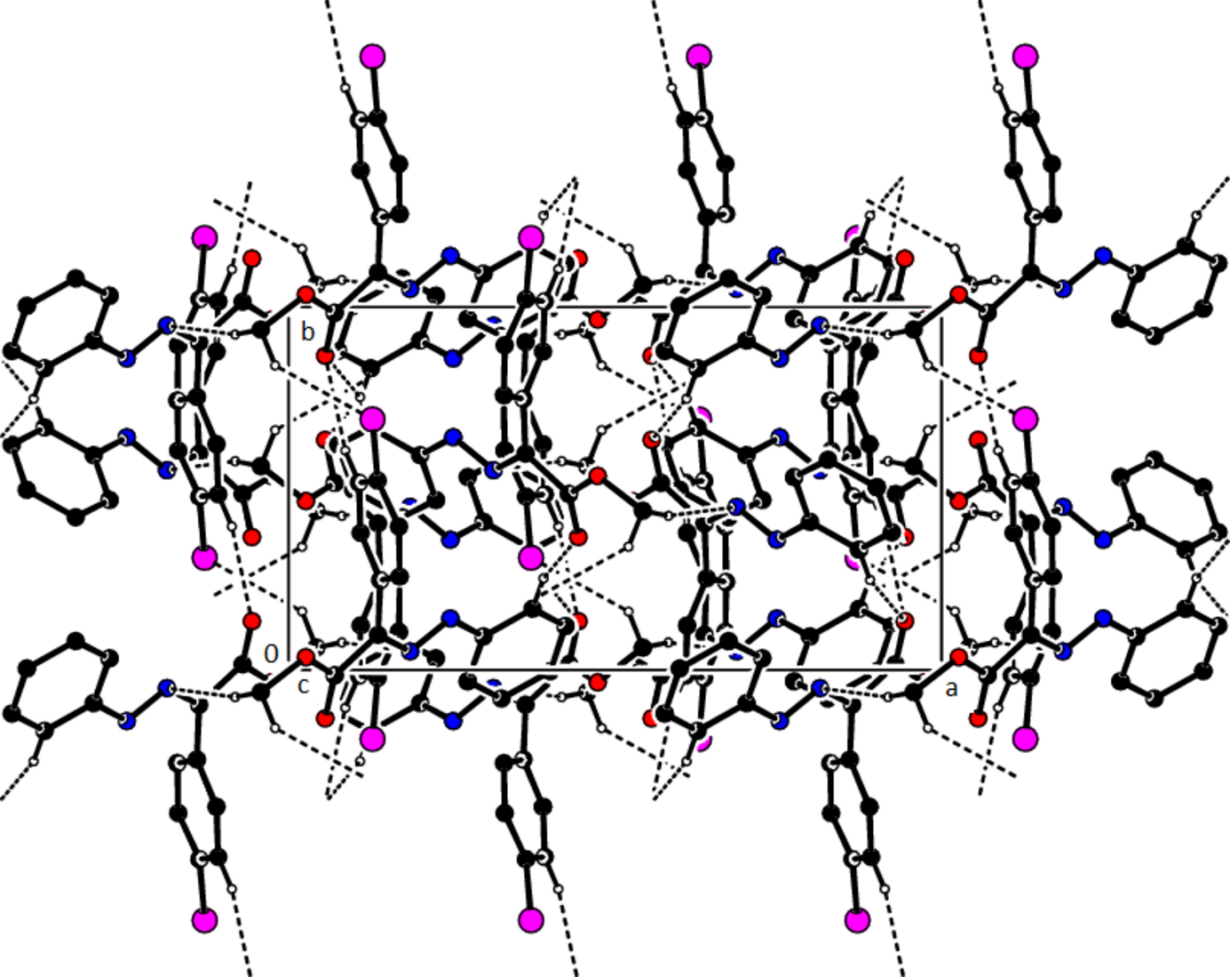
A view of the hydrogen bonds present in (**4**) in a view along the *c* axis.

**Figure 18:**
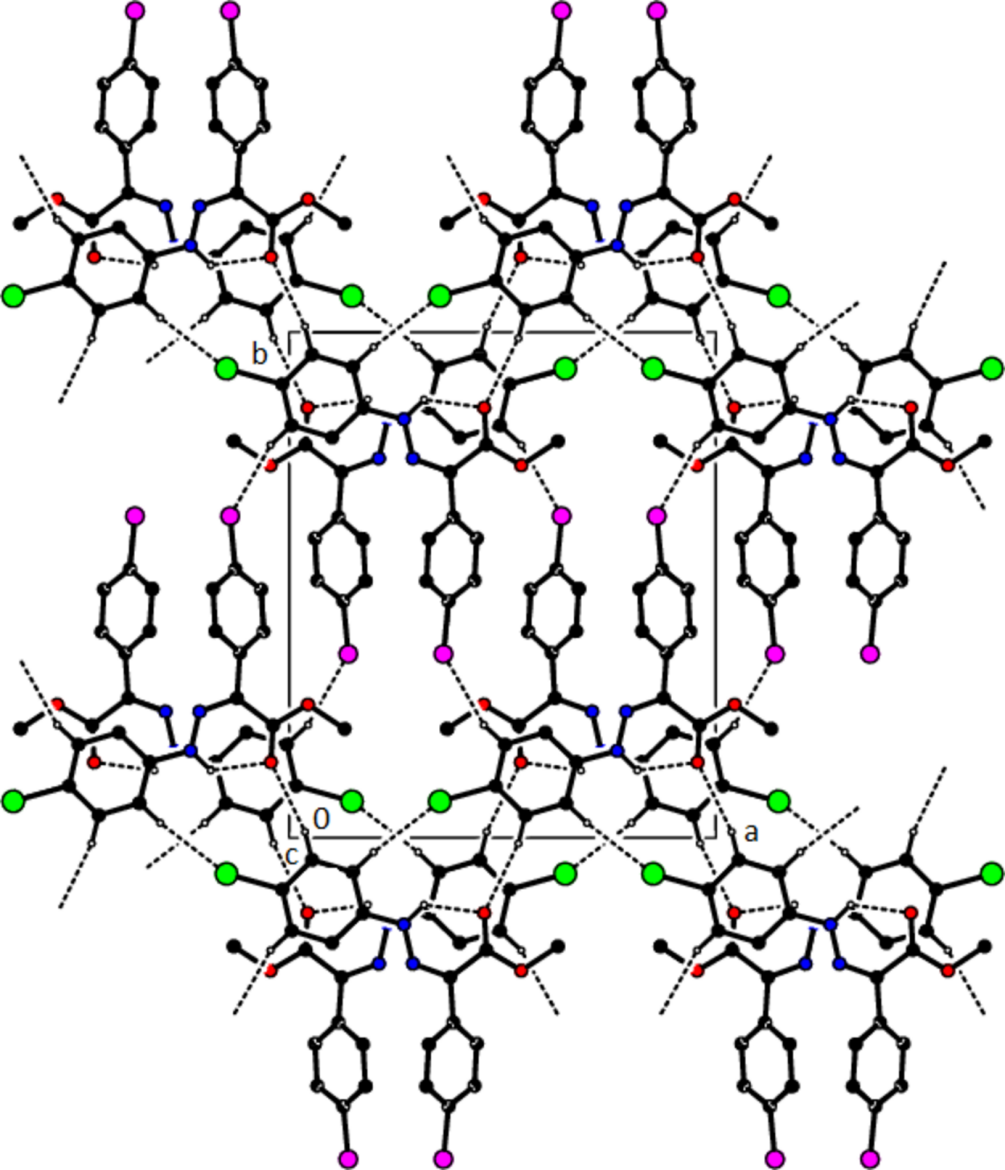
A view of the hydrogen bonds present in (**5**) in a view along the *c* axis.

**Table 1:** Hydrogen-bond geometry (Å, °) for **1**[Chem scheme1]

*D*—H⋯*A*	*D*—H	H⋯*A*	*D*⋯*A*	*D*—H⋯*A*
N2—H2⋯O1	0.95 (4)	1.93 (4)	2.668 (3)	133 (4)
C9—H9⋯O2	0.95	2.49	2.862 (6)	103
C9*A*—H9*A*⋯N1^i^	0.95	2.55	3.488 (6)	169
C5*A*—H5*A*⋯*Cg*2^ii^	0.95	2.85	3.611 (6)	138
C8—H8⋯*Cg*5^iii^	0.95	2.81	3.677 (6)	152
C8*A*—H8*A*⋯*Cg*2^i^	0.95	2.86	3.607 (6)	136
C16—H16*B*⋯*Cg*7^ii^	0.98	2.74	3.544 (3)	139

Symmetry codes: (i) 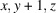; (ii) 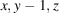; (iii) 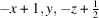.

**Table 2:** Hydrogen-bond geometry (Å, °) for **2**[Chem scheme1]

*D*—H⋯*A*	*D*—H	H⋯*A*	*D*⋯*A*	*D*—H⋯*A*
N2*A*—H2*A*⋯O1*A*	1.00 (5)	1.82 (5)	2.631 (4)	136 (4)
N2*B*—H2*B*⋯O1*B*	0.81 (5)	2.03 (5)	2.645 (4)	133 (5)
C9*A*—H9*A*⋯O2*A*	0.95	2.47	2.827 (4)	102
C9*B*—H9*B*⋯O2*B*	0.95	2.58	2.898 (4)	100
C5*A*—H5*A*⋯*Cg*3^i^	0.95	2.88	3.637 (4)	137

Symmetry code: (i) 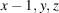.

**Table 3:** Hydrogen-bond geometry (Å, °) for **3**[Chem scheme1]

*D*—H⋯*A*	*D*—H	H⋯*A*	*D*⋯*A*	*D*—H⋯*A*
N2—H2⋯O1	0.86 (2)	1.98 (2)	2.657 (2)	134 (2)
C7—H7⋯O4^i^	0.95	2.44	3.245 (2)	142
C12—H12⋯O1^ii^	0.95	2.52	3.401 (2)	154

Symmetry codes: (i) 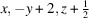; (ii) 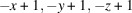.

**Table 4:** Hydrogen-bond geometry (Å, °) for **4**[Chem scheme1]

*D*—H⋯*A*	*D*—H	H⋯*A*	*D*⋯*A*	*D*—H⋯*A*
C3—H3*B*⋯N1^i^	0.98	2.55	3.5099 (15)	168
C3—H3*C*⋯Cl1^ii^	0.98	2.82	3.6422 (12)	142
C6—H6⋯O1^iii^	0.95	2.40	3.3113 (15)	161
C15—H15⋯O1^iv^	0.95	2.40	3.2759 (14)	153
C14—H14⋯*Cg*1^v^	0	2.80	3.4792 (12)	129

Symmetry codes: (i) 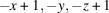; (ii) 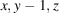; (iii) 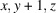; (iv) 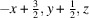; (v) 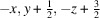.

**Table 5:** Hydrogen-bond geometry (Å, °) for **5**[Chem scheme1]

*D*—H⋯*A*	*D*—H	H⋯*A*	*D*⋯*A*	*D*—H⋯*A*
N2—H2⋯O1	0.93 (5)	2.00 (6)	2.666 (4)	127 (4)
C9—H9⋯O2	0.95	2.58	2.953 (6)	103
C11—H11⋯Br1^i^	0.95	2.75	3.689 (4)	172
C12—H12⋯O1^ii^	0.95	2.54	3.479 (4)	168
C14—H14⋯Cl1^iii^	0.95	2.70	3.616 (4)	161
C8—H8⋯*Cg*1^iv^	0.95	2.70	3.591 (6)	157

Symmetry codes: (i) 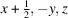; (ii) 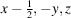; (iii) 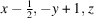; (iv) 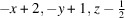.

**Table 6:** Percentage contributions of inter­atomic contacts to the Hirshfeld surface for compounds **1**, **2**, **3**, **4** and **5**

Contact	**1**	**2** *A*	**3** *B*	**3**	**4**	**5**
H⋯H	59.9	41.8	46.4	38.9	39.0	26.3
C⋯H/H⋯C	13.3	26.8	21.0	16.0	21.4	25.1
Br⋯H/H⋯Br	12.5	15.7	15.6	—	—	15.8
O⋯H/H⋯O	6.2	6.9	8.5	28.5	12.7	5.6
N⋯H/H⋯N	2.0	3.2	3.2	2.4	5.7	1.9
N⋯C/C⋯N	2.2	1.7	1.7	3.4	2.0	1.0
C⋯C	1.4	1.1	1.1	3.6	1.2	2.3
O⋯C/C⋯O	1.1	1.4	1.4	3.9	1.4	3.9
O⋯N/N⋯O	0.9	0.7	0.7	1.9	—	—
O⋯O	—	0.1	—	0.9	0.2	—
N⋯N	—	—	—	0.4	—	1.5
Br⋯O/O⋯Br	0.5	—	—	—	—	—
Br⋯C/C⋯Br	0.1	0.5	0.2	—	—	0.6
Br⋯Br	—	—	—	—	—	0.1
Cl⋯H/H⋯Cl	—	—	—	—	—	14.5
Cl⋯O/O⋯Cl	—	—	—	—	—	1.3

**Table 7:** Experimental details

	**1**	**2**	**3**	**4**	**5**
Crystal data					
Chemical formula	C_16_H_15_BrN_2_O_2_	C_17_H_17_BrN_2_O_2_	C_16_H_15_N_3_O_5_	C_15_H_13_ClN_2_O_2_	C_15_H_12_BrClN_2_O_2_
*M*_r_	347.20	361.23	329.31	288.72	367.62
Crystal system, space group	Monoclinic, *C*2/*c*	Triclinic, *P* 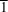	Monoclinic, *C*2/*c*	Orthorhombic, *Pbca*	Orthorhombic, *Pca*2_1_
Temperature (K)	100	100	100	100	100
*a*, *b*, *c* (Å)	34.6329 (5), 4.84061 (6), 19.1365 (3)	9.8859 (9), 12.3021 (11), 13.9790 (12)	18.8022 (5), 21.9649 (6), 7.43092 (15)	15.86326 (8), 8.79608 (3), 19.24680 (8)	14.0199 (16), 16.5940 (19), 6.4471 (9)
α, β, γ (°)	90, 109.8598 (16), 90	83.480 (9), 73.266 (7), 81.695 (8)	90, 96.156 (2), 90	90, 90, 90	90, 90, 90
*V* (Å^3^)	3017.33 (8)	1606.4 (3)	3051.19 (13)	2685.59 (2)	1499.9 (3)
*Z*	8	4	8	8	4
Radiation type	Cu *K*α	Synchrotron, λ = 0.75270 Å	Cu *K*α	Cu *K*α	Synchrotron, λ = 0.75270 Å
μ (mm^−1^)	3.77	2.97	0.91	2.55	3.35
Crystal size (mm)	0.20 × 0.05 × 0.03	0.12 × 0.09 × 0.07	0.29 × 0.10 × 0.09	0.12 × 0.11 × 0.06	0.18 × 0.15 × 0.13

Data collection					
Diffractometer	Rigaku XtaLAB Synergy-S, HyPix-6000HE area-detector	Rayonix SX165 CCD	Rigaku XtaLAB Synergy-S, HyPix-6000HE area-detector	Rigaku XtaLAB Synergy-S, HyPix-6000HE area-detector	Rayonix SX165 CCD
Absorption correction	Gaussian (*CrysAlis PRO*; Rigaku OD, 2021 ▸).	Multi-scan (*SCALA*; Evans, 2006 ▸)	Gaussian (*CrysAlis PRO*; Rigaku OD, 2021 ▸).	Multi-scan (*CrysAlis PRO*; Rigaku OD, 2021 ▸).	Multi-scan (*SCALA*; Evans, 2006 ▸)
*T*_min_, *T*_max_	0.745, 1.000	0.666, 0.789	0.322, 1.000	0.676, 1.000	0.514, 0.633
No. of measured, independent and observed [*I* > 2σ(*I*)] reflections	30865, 3282, 3031	21921, 8417, 6338	21181, 3303, 2634	51407, 2935, 2871	12123, 3908, 3677
*R*_int_	0.047	0.036	0.072	0.030	0.039
(sin θ/λ)_max_ (Å^−1^)	0.639	0.686	0.639	0.639	0.682

Refinement					
*R*[*F*^2^ > 2σ(*F*^2^)], *wR*(*F*^2^), *S*	0.043, 0.123, 1.12	0.064, 0.190, 1.07	0.046, 0.126, 1.07	0.030, 0.082, 1.08	0.040, 0.110, 1.09
No. of reflections	3282	8417	3303	2935	3908
No. of parameters	232	410	223	186	196
No. of restraints	0	0	0	0	1
H-atom treatment	H atoms treated by a mixture of independent and constrained refinement	H atoms treated by a mixture of independent and constrained refinement	H atoms treated by a mixture of independent and constrained refinement	H atoms treated by a mixture of independent and constrained refinement	H atoms treated by a mixture of independent and constrained refinement
Δρ_max_, Δρ_min_ (e Å^−3^)	1.17, −1.20	1.89, −0.94	0.27, −0.25	0.29, −0.28	1.27, −0.67
Absolute structure	–	–	–	–	Refined as an inversion twin
Absolute structure parameter	–	–	–	–	0.167 (15)

Computer programs: *CrysAlis PRO* (Rigaku OD, 2021 ▸), *Marccd* (Doyle, 2011 ▸), *iMosflm* (Battye *et al.*, 2011 ▸), *SHELXT* (Sheldrick, 2015*a* ▸), *SHELXL* (Sheldrick, 2015*b* ▸), *ORTEP-3 for Windows* (Farrugia, 2012 ▸) and *PLATON* (Spek, 2020 ▸).

## supplementary crystallographic information

### Methyl (Z)-2-(4-bromophenyl)-2-[2-(4-methylphenyl)hydrazin-1-ylidene]acetate (1) Crystal data

**Table:**

C_16_H_15_BrN_2_O_2_	*F*(000) = 1408
*M**_r_* = 347.20	*D*_x_ = 1.529 Mg m^−^^3^
Monoclinic, *C*2/*c*	Cu *K*α radiation, λ = 1.54184 Å
*a* = 34.6329 (5) Å	Cell parameters from 15931 reflections
*b* = 4.84061 (6) Å	θ = 2.7–79.1°
*c* = 19.1365 (3) Å	µ = 3.77 mm^−^^1^
β = 109.8598 (16)°	*T* = 100 K
*V* = 3017.33 (8) Å^3^	Needle, yellow
*Z* = 8	0.20 × 0.05 × 0.03 mm

### Methyl (Z)-2-(4-bromophenyl)-2-[2-(4-methylphenyl)hydrazin-1-ylidene]acetate (1) Data collection

**Table:**

Rigaku XtaLAB Synergy-S, HyPix-6000HE area-detector diffractometer	3031 reflections with *I* > 2σ(*I*)
Radiation source: micro-focus sealed X-ray tube	*R*_int_ = 0.047
φ and ω scans	θ_max_ = 80.0°, θ_min_ = 2.7°
Absorption correction: gaussian (CrysAlisPro; Rigaku OD, 2021).	*h* = −44→44
*T*_min_ = 0.745, *T*_max_ = 1.000	*k* = −6→5
30865 measured reflections	*l* = −24→24
3282 independent reflections	

### Methyl (Z)-2-(4-bromophenyl)-2-[2-(4-methylphenyl)hydrazin-1-ylidene]acetate (1) Refinement

**Table:**

Refinement on *F*^2^	Primary atom site location: difference Fourier map
Least-squares matrix: full	Secondary atom site location: difference Fourier map
*R*[*F*^2^ > 2σ(*F*^2^)] = 0.043	Hydrogen site location: mixed
*wR*(*F*^2^) = 0.123	H atoms treated by a mixture of independent and constrained refinement
*S* = 1.12	*w* = 1/[σ^2^(*F*_o_^2^) + (0.0672*P*)^2^ + 8.4257*P*] where *P* = (*F*_o_^2^ + 2*F*_c_^2^)/3
3282 reflections	(Δ/σ)_max_ = 0.003
232 parameters	Δρ_max_ = 1.17 e Å^−^^3^
0 restraints	Δρ_min_ = −1.20 e Å^−^^3^

### Methyl (Z)-2-(4-bromophenyl)-2-[2-(4-methylphenyl)hydrazin-1-ylidene]acetate (1) Special details

**Table:**

Geometry. All esds (except the esd in the dihedral angle between two l.s. planes) are estimated using the full covariance matrix. The cell esds are taken into account individually in the estimation of esds in distances, angles and torsion angles; correlations between esds in cell parameters are only used when they are defined by crystal symmetry. An approximate (isotropic) treatment of cell esds is used for estimating esds involving l.s. planes.

### Methyl (Z)-2-(4-bromophenyl)-2-[2-(4-methylphenyl)hydrazin-1-ylidene]acetate (1) Fractional atomic coordinates and isotropic or equivalent isotropic displacement parameters (Å^2^)

**Table:**

	*x*	*y*	*z*	*U*_iso_*/*U*_eq_	Occ. (<1)
Br1	0.57358 (2)	0.73045 (7)	0.43385 (2)	0.03156 (14)	
O1	0.32249 (6)	0.6995 (4)	0.20483 (12)	0.0287 (4)	
O2	0.38021 (6)	0.9149 (4)	0.20583 (10)	0.0237 (4)	
N1	0.37298 (7)	0.3715 (5)	0.32724 (12)	0.0242 (4)	
N2	0.33381 (7)	0.3087 (5)	0.30821 (13)	0.0250 (5)	
H2	0.3151 (12)	0.417 (8)	0.270 (2)	0.035 (10)*	
C1	0.38646 (8)	0.5648 (5)	0.29398 (14)	0.0218 (5)	
C2	0.35954 (9)	0.7297 (5)	0.23131 (16)	0.0229 (5)	
C3	0.35571 (9)	1.0778 (6)	0.14355 (15)	0.0288 (6)	
H3A	0.3424	0.9561	0.1013	0.043*	
H3B	0.3734	1.2099	0.1298	0.043*	
H3C	0.3347	1.1783	0.1572	0.043*	
C4	0.43162 (8)	0.6094 (5)	0.32521 (14)	0.0215 (5)	
C5	0.45024 (15)	0.6230 (11)	0.4017 (3)	0.0218 (9)	0.5
H5	0.4338	0.6105	0.4325	0.026*	0.5
C6	0.49249 (16)	0.6545 (11)	0.4344 (3)	0.0249 (10)	0.5
H6	0.5050	0.6596	0.4869	0.030*	0.5
C5A	0.45868 (17)	0.3824 (11)	0.3370 (3)	0.0261 (10)	0.5
H5A	0.4480	0.2021	0.3234	0.031*	0.5
C6A	0.50112 (17)	0.4202 (12)	0.3687 (3)	0.0268 (10)	0.5
H6A	0.5193	0.2676	0.3758	0.032*	0.5
C7	0.51588 (8)	0.6783 (6)	0.38907 (14)	0.0231 (5)	
C8	0.49822 (16)	0.6675 (12)	0.3116 (3)	0.0234 (10)	0.5
H8	0.5148	0.6812	0.2811	0.028*	0.5
C9	0.45600 (16)	0.6364 (11)	0.2801 (3)	0.0219 (9)	0.5
H9	0.4435	0.6334	0.2276	0.026*	0.5
C8A	0.49031 (19)	0.9093 (12)	0.3792 (3)	0.0315 (12)	0.5
H8A	0.5014	1.0883	0.3937	0.038*	0.5
C9A	0.44817 (18)	0.8689 (11)	0.3474 (3)	0.0293 (11)	0.5
H9A	0.4303	1.0230	0.3408	0.035*	0.5
C10	0.32137 (8)	0.1052 (5)	0.34874 (14)	0.0244 (5)	
C11	0.27995 (9)	0.0484 (6)	0.33053 (14)	0.0270 (5)	
H11	0.2603	0.1459	0.2913	0.032*	
C12	0.26724 (8)	−0.1513 (6)	0.36979 (15)	0.0251 (5)	
H12	0.2387	−0.1896	0.3563	0.030*	
C13	0.29469 (9)	−0.2977 (5)	0.42819 (15)	0.0226 (5)	
C14	0.33647 (9)	−0.2381 (5)	0.44565 (16)	0.0266 (6)	
H14	0.3561	−0.3352	0.4850	0.032*	
C15	0.34988 (8)	−0.0385 (6)	0.40626 (15)	0.0263 (5)	
H15	0.3784	−0.0012	0.4188	0.032*	
C16	0.28049 (10)	−0.5098 (6)	0.47178 (16)	0.0308 (6)	
H16A	0.2842	−0.4373	0.5214	0.046*	
H16B	0.2967	−0.6791	0.4762	0.046*	
H16C	0.2514	−0.5510	0.4460	0.046*	

### Methyl (Z)-2-(4-bromophenyl)-2-[2-(4-methylphenyl)hydrazin-1-ylidene]acetate (1) Atomic displacement parameters (Å^2^)

**Table:**

	*U*^11^	*U*^22^	*U*^33^	*U*^12^	*U*^13^	*U*^23^
Br1	0.01941 (19)	0.0424 (2)	0.02934 (19)	−0.00077 (10)	0.00364 (13)	0.00388 (11)
O1	0.0183 (9)	0.0295 (9)	0.0366 (10)	−0.0017 (7)	0.0069 (8)	0.0004 (8)
O2	0.0202 (8)	0.0263 (9)	0.0254 (8)	−0.0009 (7)	0.0087 (7)	0.0027 (7)
N1	0.0229 (10)	0.0259 (11)	0.0256 (10)	−0.0046 (9)	0.0107 (8)	−0.0049 (8)
N2	0.0222 (11)	0.0291 (11)	0.0234 (10)	−0.0026 (9)	0.0074 (9)	0.0006 (9)
C1	0.0226 (12)	0.0208 (11)	0.0234 (11)	−0.0012 (9)	0.0097 (9)	−0.0023 (9)
C2	0.0220 (13)	0.0221 (12)	0.0273 (12)	−0.0019 (9)	0.0118 (11)	−0.0031 (9)
C3	0.0290 (13)	0.0307 (13)	0.0267 (12)	0.0038 (11)	0.0092 (11)	0.0044 (11)
C4	0.0210 (12)	0.0204 (11)	0.0227 (11)	0.0005 (9)	0.0068 (9)	0.0004 (9)
C5	0.020 (2)	0.025 (2)	0.021 (2)	−0.0017 (19)	0.0077 (18)	0.0001 (18)
C6	0.023 (2)	0.026 (2)	0.022 (2)	−0.001 (2)	0.0038 (19)	0.0021 (19)
C5A	0.027 (3)	0.021 (2)	0.032 (3)	−0.002 (2)	0.011 (2)	0.000 (2)
C6A	0.028 (3)	0.026 (2)	0.031 (3)	0.003 (2)	0.015 (2)	0.006 (2)
C7	0.0188 (11)	0.0264 (12)	0.0230 (12)	−0.0007 (10)	0.0056 (9)	0.0029 (10)
C8	0.019 (2)	0.029 (2)	0.023 (2)	0.002 (2)	0.0072 (19)	0.002 (2)
C9	0.021 (2)	0.026 (2)	0.019 (2)	0.0015 (19)	0.0082 (18)	−0.0001 (18)
C8A	0.029 (3)	0.023 (2)	0.037 (3)	0.001 (2)	0.005 (2)	−0.002 (2)
C9A	0.027 (3)	0.018 (2)	0.037 (3)	0.003 (2)	0.004 (2)	−0.003 (2)
C10	0.0289 (13)	0.0249 (12)	0.0217 (11)	−0.0033 (10)	0.0115 (10)	−0.0046 (9)
C11	0.0275 (13)	0.0306 (13)	0.0218 (11)	−0.0013 (11)	0.0071 (10)	0.0009 (10)
C12	0.0200 (12)	0.0304 (13)	0.0243 (11)	−0.0030 (10)	0.0068 (10)	−0.0016 (10)
C13	0.0271 (13)	0.0198 (11)	0.0234 (12)	−0.0007 (10)	0.0117 (10)	−0.0013 (9)
C14	0.0239 (14)	0.0279 (13)	0.0269 (13)	0.0050 (10)	0.0071 (11)	−0.0028 (10)
C15	0.0202 (12)	0.0294 (13)	0.0313 (13)	−0.0036 (10)	0.0114 (10)	−0.0088 (10)
C16	0.0401 (16)	0.0237 (12)	0.0357 (14)	−0.0013 (11)	0.0222 (13)	0.0022 (11)

### Methyl (Z)-2-(4-bromophenyl)-2-[2-(4-methylphenyl)hydrazin-1-ylidene]acetate (1) Geometric parameters (Å, º)

**Table:**

Br1—C7	1.905 (3)	C6A—H6A	0.9500
O1—C2	1.218 (4)	C7—C8A	1.399 (6)
O2—C2	1.338 (3)	C7—C8	1.400 (6)
O2—C3	1.441 (3)	C8—C9	1.387 (7)
N1—C1	1.304 (3)	C8—H8	0.9500
N1—N2	1.315 (3)	C9—H9	0.9500
N2—C10	1.409 (4)	C8A—C9A	1.391 (8)
N2—H2	0.96 (4)	C8A—H8A	0.9500
C1—C2	1.478 (4)	C9A—H9A	0.9500
C1—C4	1.488 (4)	C10—C11	1.384 (4)
C3—H3A	0.9800	C10—C15	1.390 (4)
C3—H3B	0.9800	C11—C12	1.385 (4)
C3—H3C	0.9800	C11—H11	0.9500
C4—C9A	1.386 (6)	C12—C13	1.390 (4)
C4—C5	1.387 (5)	C12—H12	0.9500
C4—C9	1.403 (5)	C13—C14	1.400 (4)
C4—C5A	1.412 (6)	C13—C16	1.507 (4)
C5—C6	1.390 (7)	C14—C15	1.398 (4)
C5—H5	0.9500	C14—H14	0.9500
C6—C7	1.378 (6)	C15—H15	0.9500
C6—H6	0.9500	C16—H16A	0.9800
C5A—C6A	1.398 (8)	C16—H16B	0.9800
C5A—H5A	0.9500	C16—H16C	0.9800
C6A—C7	1.356 (6)		
			
C2—O2—C3	115.5 (2)	C8A—C7—Br1	118.3 (3)
C1—N1—N2	122.5 (2)	C8—C7—Br1	119.7 (3)
N1—N2—C10	119.3 (2)	C9—C8—C7	118.7 (4)
N1—N2—H2	117 (2)	C9—C8—H8	120.6
C10—N2—H2	124 (2)	C7—C8—H8	120.6
N1—C1—C2	123.5 (2)	C8—C9—C4	120.6 (4)
N1—C1—C4	114.2 (2)	C8—C9—H9	119.7
C2—C1—C4	122.3 (2)	C4—C9—H9	119.7
O1—C2—O2	123.2 (3)	C9A—C8A—C7	118.0 (5)
O1—C2—C1	123.9 (2)	C9A—C8A—H8A	121.0
O2—C2—C1	112.9 (2)	C7—C8A—H8A	121.0
O2—C3—H3A	109.5	C4—C9A—C8A	121.6 (5)
O2—C3—H3B	109.5	C4—C9A—H9A	119.2
H3A—C3—H3B	109.5	C8A—C9A—H9A	119.2
O2—C3—H3C	109.5	C11—C10—C15	119.8 (2)
H3A—C3—H3C	109.5	C11—C10—N2	119.0 (2)
H3B—C3—H3C	109.5	C15—C10—N2	121.2 (2)
C5—C4—C9	118.9 (3)	C10—C11—C12	119.7 (3)
C9A—C4—C5A	118.2 (4)	C10—C11—H11	120.1
C9A—C4—C1	121.6 (3)	C12—C11—H11	120.1
C5—C4—C1	118.6 (3)	C11—C12—C13	122.4 (2)
C9—C4—C1	122.4 (3)	C11—C12—H12	118.8
C5A—C4—C1	120.1 (3)	C13—C12—H12	118.8
C4—C5—C6	121.4 (4)	C12—C13—C14	117.1 (2)
C4—C5—H5	119.3	C12—C13—C16	122.0 (3)
C6—C5—H5	119.3	C14—C13—C16	120.9 (3)
C7—C6—C5	118.7 (4)	C15—C14—C13	121.3 (3)
C7—C6—H6	120.7	C15—C14—H14	119.4
C5—C6—H6	120.7	C13—C14—H14	119.4
C6A—C5A—C4	120.7 (5)	C10—C15—C14	119.7 (2)
C6A—C5A—H5A	119.6	C10—C15—H15	120.1
C4—C5A—H5A	119.6	C14—C15—H15	120.1
C7—C6A—C5A	118.9 (5)	C13—C16—H16A	109.5
C7—C6A—H6A	120.6	C13—C16—H16B	109.5
C5A—C6A—H6A	120.6	H16A—C16—H16B	109.5
C6A—C7—C8A	122.6 (4)	C13—C16—H16C	109.5
C6—C7—C8	121.7 (4)	H16A—C16—H16C	109.5
C6A—C7—Br1	119.1 (3)	H16B—C16—H16C	109.5
C6—C7—Br1	118.6 (3)		
			
C1—N1—N2—C10	−177.4 (2)	C5—C6—C7—C8	−0.9 (7)
N2—N1—C1—C2	−0.9 (4)	C5—C6—C7—Br1	178.5 (4)
N2—N1—C1—C4	178.3 (2)	C6A—C7—C8—C9	68.3 (5)
C3—O2—C2—O1	−0.6 (4)	C6—C7—C8—C9	1.0 (7)
C3—O2—C2—C1	178.6 (2)	C8A—C7—C8—C9	−60.4 (5)
N1—C1—C2—O1	−0.4 (4)	Br1—C7—C8—C9	−178.4 (4)
C4—C1—C2—O1	−179.5 (3)	C7—C8—C9—C4	−1.6 (8)
N1—C1—C2—O2	−179.7 (2)	C9A—C4—C9—C8	61.1 (5)
C4—C1—C2—O2	1.2 (3)	C5—C4—C9—C8	2.0 (7)
N1—C1—C4—C9A	−126.8 (4)	C5A—C4—C9—C8	−63.7 (5)
C2—C1—C4—C9A	52.4 (4)	C1—C4—C9—C8	−177.6 (4)
N1—C1—C4—C5	−45.3 (4)	C6A—C7—C8A—C9A	0.3 (8)
C2—C1—C4—C5	133.9 (3)	C6—C7—C8A—C9A	−66.2 (6)
N1—C1—C4—C9	134.2 (3)	C8—C7—C8A—C9A	61.5 (6)
C2—C1—C4—C9	−46.6 (4)	Br1—C7—C8A—C9A	−179.1 (4)
N1—C1—C4—C5A	49.4 (4)	C5—C4—C9A—C8A	66.2 (6)
C2—C1—C4—C5A	−131.4 (4)	C9—C4—C9A—C8A	−60.2 (6)
C9A—C4—C5—C6	−67.0 (5)	C5A—C4—C9A—C8A	1.3 (8)
C9—C4—C5—C6	−1.9 (7)	C1—C4—C9A—C8A	177.6 (5)
C5A—C4—C5—C6	58.9 (5)	C7—C8A—C9A—C4	−0.7 (9)
C1—C4—C5—C6	177.7 (4)	N1—N2—C10—C11	177.2 (2)
C4—C5—C6—C7	1.3 (8)	N1—N2—C10—C15	−2.8 (4)
C9A—C4—C5A—C6A	−1.5 (7)	C15—C10—C11—C12	0.0 (4)
C5—C4—C5A—C6A	−60.5 (5)	N2—C10—C11—C12	179.9 (2)
C9—C4—C5A—C6A	65.3 (5)	C10—C11—C12—C13	0.7 (4)
C1—C4—C5A—C6A	−177.8 (4)	C11—C12—C13—C14	−0.9 (4)
C4—C5A—C6A—C7	1.1 (7)	C11—C12—C13—C16	178.5 (3)
C5A—C6A—C7—C6	60.5 (5)	C12—C13—C14—C15	0.4 (4)
C5A—C6A—C7—C8A	−0.5 (7)	C16—C13—C14—C15	−179.0 (2)
C5A—C6A—C7—C8	−67.0 (5)	C11—C10—C15—C14	−0.4 (4)
C5A—C6A—C7—Br1	178.9 (4)	N2—C10—C15—C14	179.6 (2)
C5—C6—C7—C6A	−62.6 (5)	C13—C14—C15—C10	0.2 (4)
C5—C6—C7—C8A	66.0 (5)		

### Methyl (Z)-2-(4-bromophenyl)-2-[2-(4-methylphenyl)hydrazin-1-ylidene]acetate (1) Hydrogen-bond geometry (Å, º)

**Table:**

*D*—H···*A*	*D*—H	H···*A*	*D*···*A*	*D*—H···*A*
N2—H2···O1	0.95 (4)	1.93 (4)	2.668 (3)	133 (4)
C9—H9···O2	0.95	2.49	2.862 (6)	103
C9*A*—H9*A*···N1^i^	0.95	2.55	3.488 (6)	169
C5*A*—H5*A*···*Cg*2^ii^	0.95	2.85	3.611 (6)	138
C8—H8···*Cg*5^iii^	0.95	2.81	3.677 (6)	152
C8*A*—H8*A*···*Cg*2^i^	0.95	2.86	3.607 (6)	136
C16—H16*B*···*Cg*7^ii^	0.98	2.74	3.544 (3)	139

Symmetry codes: (i) *x*, *y*+1, *z*; (ii) *x*, *y*−1, *z*; (iii) −*x*+1, *y*, −*z*+1/2.

### Methyl (Z)-2-(4-bromophenyl)-2-[2-(3,5-dimethylphenyl)hydrazin-1-ylidene]acetate (2) Crystal data

**Table:**

C_17_H_17_BrN_2_O_2_	*Z* = 4
*M**_r_* = 361.23	*F*(000) = 736
Triclinic, *P*1	*D*_x_ = 1.494 Mg m^−^^3^
*a* = 9.8859 (9) Å	Synchrotron radiation, λ = 0.75270 Å
*b* = 12.3021 (11) Å	Cell parameters from 1000 reflections
*c* = 13.9790 (12) Å	θ = 1.8–30.0°
α = 83.480 (9)°	µ = 2.97 mm^−^^1^
β = 73.266 (7)°	*T* = 100 K
γ = 81.695 (8)°	Prism, yellow
*V* = 1606.4 (3) Å^3^	0.12 × 0.09 × 0.07 mm

### Methyl (Z)-2-(4-bromophenyl)-2-[2-(3,5-dimethylphenyl)hydrazin-1-ylidene]acetate (2) Data collection

**Table:**

Rayonix SX165 CCD diffractometer	6338 reflections with *I* > 2σ(*I*)
/f scan	*R*_int_ = 0.036
Absorption correction: multi-scan (Scala; Evans, 2006)	θ_max_ = 31.1°, θ_min_ = 1.8°
*T*_min_ = 0.666, *T*_max_ = 0.789	*h* = −13→13
21921 measured reflections	*k* = −16→16
8417 independent reflections	*l* = −18→19

### Methyl (Z)-2-(4-bromophenyl)-2-[2-(3,5-dimethylphenyl)hydrazin-1-ylidene]acetate (2) Refinement

**Table:**

Refinement on *F*^2^	Hydrogen site location: mixed
Least-squares matrix: full	H atoms treated by a mixture of independent and constrained refinement
*R*[*F*^2^ > 2σ(*F*^2^)] = 0.064	*w* = 1/[σ^2^(*F*_o_^2^) + (0.1079*P*)^2^ + 1.6265*P*] where *P* = (*F*_o_^2^ + 2*F*_c_^2^)/3
*wR*(*F*^2^) = 0.190	(Δ/σ)_max_ < 0.001
*S* = 1.07	Δρ_max_ = 1.89 e Å^−^^3^
8417 reflections	Δρ_min_ = −0.94 e Å^−^^3^
410 parameters	Extinction correction: SHELXL-2019/2 (Sheldrick, 2015a), Fc^*^=kFc[1+0.001xFc^2^λ^3^/sin(2θ)]^-1/4^
0 restraints	Extinction coefficient: 0.044 (3)

### Methyl (Z)-2-(4-bromophenyl)-2-[2-(3,5-dimethylphenyl)hydrazin-1-ylidene]acetate (2) Special details

**Table:**

Geometry. All esds (except the esd in the dihedral angle between two l.s. planes) are estimated using the full covariance matrix. The cell esds are taken into account individually in the estimation of esds in distances, angles and torsion angles; correlations between esds in cell parameters are only used when they are defined by crystal symmetry. An approximate (isotropic) treatment of cell esds is used for estimating esds involving l.s. planes.

### Methyl (Z)-2-(4-bromophenyl)-2-[2-(3,5-dimethylphenyl)hydrazin-1-ylidene]acetate (2) Fractional atomic coordinates and isotropic or equivalent isotropic displacement parameters (Å^2^)

**Table:**

	*x*	*y*	*z*	*U*_iso_*/*U*_eq_	
C1A	0.4335 (3)	0.3106 (3)	0.6750 (3)	0.0279 (6)	
C2A	0.4825 (3)	0.4119 (3)	0.6932 (3)	0.0294 (7)	
C3A	0.6205 (4)	0.5560 (3)	0.6248 (3)	0.0423 (9)	
H3AA	0.677439	0.585644	0.560006	0.063*	
H3AB	0.677900	0.543397	0.672504	0.063*	
H3AC	0.536763	0.608779	0.650156	0.063*	
C4A	0.5061 (3)	0.2504 (3)	0.5848 (2)	0.0261 (6)	
C5A	0.4260 (3)	0.1991 (3)	0.5392 (3)	0.0316 (7)	
H5A	0.325686	0.203652	0.566444	0.038*	
C6A	0.4906 (4)	0.1420 (3)	0.4553 (3)	0.0319 (7)	
H6A	0.435177	0.106993	0.425294	0.038*	
C7A	0.6361 (4)	0.1361 (3)	0.4152 (3)	0.0301 (7)	
C8A	0.7196 (3)	0.1832 (3)	0.4598 (3)	0.0318 (7)	
H8A	0.819991	0.177166	0.432725	0.038*	
C9A	0.6534 (3)	0.2393 (3)	0.5451 (3)	0.0309 (7)	
H9A	0.709808	0.270754	0.576918	0.037*	
C10A	0.1256 (4)	0.2743 (3)	0.8804 (3)	0.0316 (7)	
C11A	0.0567 (4)	0.3239 (3)	0.9683 (3)	0.0369 (8)	
H11A	0.092046	0.385259	0.984307	0.044*	
C12A	−0.0639 (4)	0.2842 (3)	1.0332 (3)	0.0375 (8)	
C13A	−0.1141 (4)	0.1949 (3)	1.0083 (3)	0.0327 (7)	
H13A	−0.197025	0.168028	1.052340	0.039*	
C14A	−0.0465 (4)	0.1436 (3)	0.9208 (3)	0.0307 (7)	
C15A	0.0755 (4)	0.1845 (3)	0.8550 (3)	0.0314 (7)	
H15A	0.122838	0.151241	0.794216	0.038*	
C16A	−0.1391 (5)	0.3384 (4)	1.1287 (3)	0.0500 (11)	
H16C	−0.236055	0.368284	1.127359	0.075*	
H16D	−0.087377	0.398205	1.134887	0.075*	
H16E	−0.142998	0.283830	1.185948	0.075*	
C17A	−0.1000 (4)	0.0467 (3)	0.8954 (3)	0.0363 (8)	
H17C	−0.070369	−0.019532	0.933805	0.054*	
H17D	−0.060773	0.037247	0.823594	0.054*	
H17E	−0.204056	0.058564	0.912015	0.054*	
Br1A	0.72473 (4)	0.06195 (3)	0.29756 (3)	0.03885 (15)	
N1A	0.3195 (3)	0.2721 (2)	0.7342 (2)	0.0305 (6)	
N2A	0.2473 (3)	0.3176 (3)	0.8175 (2)	0.0318 (6)	
O1A	0.4383 (3)	0.4574 (2)	0.7710 (2)	0.0333 (5)	
O2A	0.5755 (3)	0.4536 (2)	0.6125 (2)	0.0331 (5)	
H2A	0.294 (5)	0.376 (4)	0.835 (4)	0.040*	
C1B	0.9468 (3)	0.3394 (3)	0.6648 (2)	0.0260 (6)	
C2B	1.0061 (3)	0.4347 (3)	0.6847 (2)	0.0262 (6)	
C3B	1.1954 (4)	0.5434 (3)	0.6364 (3)	0.0322 (7)	
H3BA	1.285785	0.549359	0.584772	0.048*	
H3BB	1.212847	0.530187	0.702487	0.048*	
H3BC	1.131891	0.612029	0.633975	0.048*	
C4B	1.0166 (3)	0.2763 (3)	0.5762 (2)	0.0258 (6)	
C5B	1.0352 (3)	0.1616 (3)	0.5884 (3)	0.0282 (6)	
H5B	1.003525	0.125356	0.653181	0.034*	
C6B	1.0992 (3)	0.1001 (3)	0.5070 (3)	0.0303 (7)	
H6B	1.114012	0.022028	0.516203	0.036*	
C7B	1.1416 (3)	0.1523 (3)	0.4120 (3)	0.0283 (6)	
C8B	1.1236 (3)	0.2656 (3)	0.3969 (3)	0.0300 (7)	
H8B	1.152827	0.301006	0.331502	0.036*	
C9B	1.0619 (3)	0.3266 (3)	0.4793 (3)	0.0283 (6)	
H9B	1.049927	0.404698	0.469699	0.034*	
C10B	0.6466 (3)	0.2975 (3)	0.8746 (3)	0.0275 (6)	
C11B	0.5797 (4)	0.3428 (3)	0.9656 (3)	0.0298 (7)	
H11B	0.613998	0.404242	0.982436	0.036*	
C12B	0.4637 (4)	0.2990 (3)	1.0317 (3)	0.0328 (7)	
C13B	0.4136 (4)	0.2099 (3)	1.0046 (3)	0.0316 (7)	
H13B	0.333141	0.180104	1.049162	0.038*	
C14B	0.4789 (3)	0.1636 (3)	0.9135 (3)	0.0295 (7)	
C15B	0.5967 (3)	0.2091 (3)	0.8484 (2)	0.0277 (6)	
H15B	0.642570	0.178951	0.785965	0.033*	
C16B	0.3916 (5)	0.3463 (4)	1.1314 (3)	0.0427 (9)	
H16F	0.291085	0.370073	1.135972	0.064*	
H16G	0.437852	0.409619	1.137109	0.064*	
H16H	0.399070	0.289955	1.185660	0.064*	
C17B	0.4269 (4)	0.0673 (3)	0.8860 (3)	0.0342 (7)	
H17F	0.451926	0.067228	0.812845	0.051*	
H17G	0.323359	0.071899	0.913332	0.051*	
H17H	0.471205	−0.000818	0.913587	0.051*	
Br1B	1.22812 (4)	0.06754 (3)	0.30080 (3)	0.03961 (16)	
N1B	0.8342 (3)	0.3006 (2)	0.7260 (2)	0.0275 (5)	
N2B	0.7636 (3)	0.3450 (2)	0.8103 (2)	0.0278 (6)	
O1B	0.9482 (3)	0.4914 (2)	0.7546 (2)	0.0334 (5)	
O2B	1.1299 (2)	0.45294 (19)	0.61850 (18)	0.0279 (5)	
H2B	0.793 (5)	0.396 (4)	0.827 (4)	0.034*	

### Methyl (Z)-2-(4-bromophenyl)-2-[2-(3,5-dimethylphenyl)hydrazin-1-ylidene]acetate (2) Atomic displacement parameters (Å^2^)

**Table:**

	*U*^11^	*U*^22^	*U*^33^	*U*^12^	*U*^13^	*U*^23^
C1A	0.0274 (14)	0.0292 (15)	0.0269 (16)	−0.0017 (11)	−0.0068 (12)	−0.0047 (12)
C2A	0.0272 (14)	0.0316 (16)	0.0290 (17)	−0.0008 (12)	−0.0074 (12)	−0.0056 (13)
C3A	0.050 (2)	0.0344 (19)	0.039 (2)	−0.0136 (16)	0.0002 (17)	−0.0116 (16)
C4A	0.0258 (14)	0.0258 (14)	0.0259 (16)	−0.0016 (11)	−0.0065 (11)	−0.0022 (12)
C5A	0.0266 (14)	0.0354 (17)	0.0334 (18)	−0.0009 (12)	−0.0097 (13)	−0.0055 (14)
C6A	0.0316 (16)	0.0342 (17)	0.0315 (18)	−0.0042 (12)	−0.0092 (13)	−0.0074 (14)
C7A	0.0365 (16)	0.0252 (15)	0.0263 (16)	0.0007 (12)	−0.0060 (13)	−0.0045 (12)
C8A	0.0264 (14)	0.0255 (15)	0.041 (2)	−0.0021 (11)	−0.0049 (13)	−0.0054 (13)
C9A	0.0304 (15)	0.0268 (15)	0.0366 (19)	−0.0048 (12)	−0.0088 (13)	−0.0063 (13)
C10A	0.0292 (15)	0.0319 (16)	0.0299 (17)	0.0010 (12)	−0.0047 (13)	−0.0017 (13)
C11A	0.0404 (18)	0.0351 (18)	0.0331 (19)	−0.0020 (14)	−0.0065 (15)	−0.0062 (15)
C12A	0.0423 (19)	0.0365 (18)	0.0295 (18)	−0.0017 (14)	−0.0037 (14)	−0.0054 (14)
C13A	0.0298 (15)	0.0377 (18)	0.0260 (17)	0.0002 (13)	−0.0030 (12)	−0.0015 (13)
C14A	0.0307 (15)	0.0310 (16)	0.0293 (17)	0.0008 (12)	−0.0089 (13)	−0.0011 (13)
C15A	0.0318 (16)	0.0364 (17)	0.0237 (16)	0.0039 (13)	−0.0071 (12)	−0.0048 (13)
C16A	0.053 (2)	0.052 (2)	0.036 (2)	−0.0084 (19)	0.0076 (18)	−0.0136 (19)
C17A	0.0341 (17)	0.0366 (18)	0.036 (2)	−0.0039 (14)	−0.0060 (14)	−0.0038 (15)
Br1A	0.0446 (2)	0.0352 (2)	0.0327 (2)	0.00094 (15)	−0.00333 (16)	−0.01194 (15)
N1A	0.0312 (13)	0.0321 (14)	0.0267 (14)	0.0011 (11)	−0.0079 (11)	−0.0022 (11)
N2A	0.0325 (14)	0.0310 (14)	0.0303 (15)	−0.0042 (11)	−0.0048 (11)	−0.0056 (12)
O1A	0.0336 (12)	0.0338 (13)	0.0314 (13)	−0.0013 (9)	−0.0064 (10)	−0.0086 (10)
O2A	0.0379 (13)	0.0268 (11)	0.0324 (13)	−0.0061 (9)	−0.0033 (10)	−0.0070 (10)
C1B	0.0259 (13)	0.0263 (14)	0.0249 (15)	−0.0037 (11)	−0.0046 (11)	−0.0029 (12)
C2B	0.0262 (14)	0.0286 (15)	0.0222 (15)	−0.0003 (11)	−0.0052 (11)	−0.0026 (12)
C3B	0.0317 (16)	0.0310 (16)	0.0339 (18)	−0.0067 (12)	−0.0069 (13)	−0.0045 (13)
C4B	0.0236 (13)	0.0267 (15)	0.0253 (15)	−0.0017 (11)	−0.0031 (11)	−0.0058 (12)
C5B	0.0285 (14)	0.0270 (15)	0.0284 (16)	−0.0043 (11)	−0.0061 (12)	−0.0021 (12)
C6B	0.0322 (15)	0.0244 (14)	0.0344 (18)	−0.0050 (11)	−0.0078 (13)	−0.0052 (13)
C7B	0.0277 (14)	0.0316 (16)	0.0254 (16)	−0.0034 (12)	−0.0034 (12)	−0.0111 (13)
C8B	0.0315 (15)	0.0322 (16)	0.0257 (16)	−0.0062 (12)	−0.0050 (12)	−0.0041 (13)
C9B	0.0278 (14)	0.0275 (15)	0.0262 (16)	0.0000 (11)	−0.0033 (12)	−0.0029 (12)
C10B	0.0256 (14)	0.0303 (15)	0.0238 (16)	0.0015 (11)	−0.0039 (11)	−0.0038 (12)
C11B	0.0322 (15)	0.0296 (16)	0.0252 (16)	−0.0035 (12)	−0.0032 (12)	−0.0052 (12)
C12B	0.0326 (16)	0.0347 (17)	0.0253 (17)	−0.0005 (13)	0.0003 (13)	−0.0045 (13)
C13B	0.0296 (15)	0.0328 (17)	0.0268 (17)	−0.0023 (12)	−0.0002 (12)	−0.0008 (13)
C14B	0.0274 (14)	0.0282 (15)	0.0317 (17)	−0.0011 (11)	−0.0080 (12)	−0.0004 (13)
C15B	0.0279 (14)	0.0296 (15)	0.0218 (15)	0.0013 (11)	−0.0027 (11)	−0.0032 (12)
C16B	0.046 (2)	0.047 (2)	0.0292 (19)	−0.0101 (17)	0.0057 (15)	−0.0131 (16)
C17B	0.0324 (16)	0.0307 (17)	0.039 (2)	−0.0043 (13)	−0.0076 (14)	−0.0062 (14)
Br1B	0.0464 (2)	0.0367 (2)	0.0340 (2)	−0.00589 (15)	−0.00226 (16)	−0.01727 (16)
N1B	0.0269 (12)	0.0299 (13)	0.0246 (14)	−0.0008 (10)	−0.0057 (10)	−0.0046 (11)
N2B	0.0276 (13)	0.0308 (14)	0.0228 (14)	−0.0024 (10)	−0.0020 (10)	−0.0079 (11)
O1B	0.0344 (12)	0.0319 (12)	0.0314 (13)	−0.0052 (9)	−0.0025 (10)	−0.0084 (10)
O2B	0.0272 (11)	0.0292 (11)	0.0266 (12)	−0.0049 (8)	−0.0042 (9)	−0.0052 (9)

### Methyl (Z)-2-(4-bromophenyl)-2-[2-(3,5-dimethylphenyl)hydrazin-1-ylidene]acetate (2) Geometric parameters (Å, º)

**Table:**

C1A—N1A	1.307 (4)	C1B—N1B	1.308 (4)
C1A—C2A	1.473 (5)	C1B—C2B	1.471 (4)
C1A—C4A	1.480 (5)	C1B—C4B	1.478 (4)
C2A—O1A	1.217 (4)	C2B—O1B	1.219 (4)
C2A—O2A	1.337 (4)	C2B—O2B	1.336 (4)
C3A—O2A	1.439 (4)	C3B—O2B	1.444 (4)
C3A—H3AA	0.9800	C3B—H3BA	0.9800
C3A—H3AB	0.9800	C3B—H3BB	0.9800
C3A—H3AC	0.9800	C3B—H3BC	0.9800
C4A—C9A	1.394 (4)	C4B—C5B	1.394 (4)
C4A—C5A	1.399 (4)	C4B—C9B	1.400 (5)
C5A—C6A	1.380 (5)	C5B—C6B	1.382 (5)
C5A—H5A	0.9500	C5B—H5B	0.9500
C6A—C7A	1.380 (5)	C6B—C7B	1.383 (5)
C6A—H6A	0.9500	C6B—H6B	0.9500
C7A—C8A	1.387 (5)	C7B—C8B	1.379 (5)
C7A—Br1A	1.893 (3)	C7B—Br1B	1.893 (3)
C8A—C9A	1.390 (5)	C8B—C9B	1.386 (4)
C8A—H8A	0.9500	C8B—H8B	0.9500
C9A—H9A	0.9500	C9B—H9B	0.9500
C10A—C11A	1.384 (5)	C10B—C15B	1.382 (5)
C10A—C15A	1.393 (5)	C10B—C11B	1.391 (5)
C10A—N2A	1.405 (4)	C10B—N2B	1.402 (4)
C11A—C12A	1.388 (5)	C11B—C12B	1.384 (5)
C11A—H11A	0.9500	C11B—H11B	0.9500
C12A—C13A	1.385 (5)	C12B—C13B	1.396 (5)
C12A—C16A	1.505 (5)	C12B—C16B	1.510 (5)
C13A—C14A	1.387 (5)	C13B—C14B	1.396 (5)
C13A—H13A	0.9500	C13B—H13B	0.9500
C14A—C15A	1.409 (5)	C14B—C15B	1.399 (5)
C14A—C17A	1.487 (5)	C14B—C17B	1.487 (5)
C15A—H15A	0.9500	C15B—H15B	0.9500
C16A—H16C	0.9800	C16B—H16F	0.9800
C16A—H16D	0.9800	C16B—H16G	0.9800
C16A—H16E	0.9800	C16B—H16H	0.9800
C17A—H17C	0.9800	C17B—H17F	0.9800
C17A—H17D	0.9800	C17B—H17G	0.9800
C17A—H17E	0.9800	C17B—H17H	0.9800
N1A—N2A	1.316 (4)	N1B—N2B	1.317 (4)
N2A—H2A	0.99 (5)	N2B—H2B	0.81 (5)
			
N1A—C1A—C2A	122.2 (3)	N1B—C1B—C2B	123.0 (3)
N1A—C1A—C4A	116.2 (3)	N1B—C1B—C4B	115.2 (3)
C2A—C1A—C4A	121.6 (3)	C2B—C1B—C4B	121.7 (3)
O1A—C2A—O2A	122.7 (3)	O1B—C2B—O2B	123.4 (3)
O1A—C2A—C1A	124.4 (3)	O1B—C2B—C1B	123.4 (3)
O2A—C2A—C1A	112.9 (3)	O2B—C2B—C1B	113.2 (3)
O2A—C3A—H3AA	109.5	O2B—C3B—H3BA	109.5
O2A—C3A—H3AB	109.5	O2B—C3B—H3BB	109.5
H3AA—C3A—H3AB	109.5	H3BA—C3B—H3BB	109.5
O2A—C3A—H3AC	109.5	O2B—C3B—H3BC	109.5
H3AA—C3A—H3AC	109.5	H3BA—C3B—H3BC	109.5
H3AB—C3A—H3AC	109.5	H3BB—C3B—H3BC	109.5
C9A—C4A—C5A	118.2 (3)	C5B—C4B—C9B	118.0 (3)
C9A—C4A—C1A	122.2 (3)	C5B—C4B—C1B	119.1 (3)
C5A—C4A—C1A	119.6 (3)	C9B—C4B—C1B	122.8 (3)
C6A—C5A—C4A	120.9 (3)	C6B—C5B—C4B	120.6 (3)
C6A—C5A—H5A	119.5	C6B—C5B—H5B	119.7
C4A—C5A—H5A	119.5	C4B—C5B—H5B	119.7
C5A—C6A—C7A	119.6 (3)	C5B—C6B—C7B	119.9 (3)
C5A—C6A—H6A	120.2	C5B—C6B—H6B	120.0
C7A—C6A—H6A	120.2	C7B—C6B—H6B	120.0
C6A—C7A—C8A	121.1 (3)	C8B—C7B—C6B	121.1 (3)
C6A—C7A—Br1A	119.8 (3)	C8B—C7B—Br1B	119.1 (3)
C8A—C7A—Br1A	119.0 (3)	C6B—C7B—Br1B	119.7 (3)
C7A—C8A—C9A	118.7 (3)	C7B—C8B—C9B	118.4 (3)
C7A—C8A—H8A	120.7	C7B—C8B—H8B	120.8
C9A—C8A—H8A	120.7	C9B—C8B—H8B	120.8
C8A—C9A—C4A	121.3 (3)	C8B—C9B—C4B	121.8 (3)
C8A—C9A—H9A	119.3	C8B—C9B—H9B	119.1
C4A—C9A—H9A	119.3	C4B—C9B—H9B	119.1
C11A—C10A—C15A	121.0 (3)	C15B—C10B—C11B	120.4 (3)
C11A—C10A—N2A	117.9 (3)	C15B—C10B—N2B	121.4 (3)
C15A—C10A—N2A	121.1 (3)	C11B—C10B—N2B	118.2 (3)
C10A—C11A—C12A	120.2 (4)	C12B—C11B—C10B	120.5 (3)
C10A—C11A—H11A	119.9	C12B—C11B—H11B	119.7
C12A—C11A—H11A	119.9	C10B—C11B—H11B	119.7
C13A—C12A—C11A	119.0 (3)	C11B—C12B—C13B	118.7 (3)
C13A—C12A—C16A	120.7 (4)	C11B—C12B—C16B	121.0 (3)
C11A—C12A—C16A	120.3 (4)	C13B—C12B—C16B	120.2 (3)
C12A—C13A—C14A	121.8 (3)	C14B—C13B—C12B	121.6 (3)
C12A—C13A—H13A	119.1	C14B—C13B—H13B	119.2
C14A—C13A—H13A	119.1	C12B—C13B—H13B	119.2
C13A—C14A—C15A	119.0 (3)	C13B—C14B—C15B	118.4 (3)
C13A—C14A—C17A	121.4 (3)	C13B—C14B—C17B	121.3 (3)
C15A—C14A—C17A	119.6 (3)	C15B—C14B—C17B	120.3 (3)
C10A—C15A—C14A	119.0 (3)	C10B—C15B—C14B	120.4 (3)
C10A—C15A—H15A	120.5	C10B—C15B—H15B	119.8
C14A—C15A—H15A	120.5	C14B—C15B—H15B	119.8
C12A—C16A—H16C	109.5	C12B—C16B—H16F	109.5
C12A—C16A—H16D	109.5	C12B—C16B—H16G	109.5
H16C—C16A—H16D	109.5	H16F—C16B—H16G	109.5
C12A—C16A—H16E	109.5	C12B—C16B—H16H	109.5
H16C—C16A—H16E	109.5	H16F—C16B—H16H	109.5
H16D—C16A—H16E	109.5	H16G—C16B—H16H	109.5
C14A—C17A—H17C	109.5	C14B—C17B—H17F	109.5
C14A—C17A—H17D	109.5	C14B—C17B—H17G	109.5
H17C—C17A—H17D	109.5	H17F—C17B—H17G	109.5
C14A—C17A—H17E	109.5	C14B—C17B—H17H	109.5
H17C—C17A—H17E	109.5	H17F—C17B—H17H	109.5
H17D—C17A—H17E	109.5	H17G—C17B—H17H	109.5
C1A—N1A—N2A	121.7 (3)	C1B—N1B—N2B	122.5 (3)
N1A—N2A—C10A	120.6 (3)	N1B—N2B—C10B	119.9 (3)
N1A—N2A—H2A	114 (3)	N1B—N2B—H2B	120 (3)
C10A—N2A—H2A	125 (3)	C10B—N2B—H2B	120 (3)
C2A—O2A—C3A	115.0 (3)	C2B—O2B—C3B	115.6 (3)
			
N1A—C1A—C2A—O1A	13.0 (5)	N1B—C1B—C2B—O1B	−7.1 (5)
C4A—C1A—C2A—O1A	−170.9 (3)	C4B—C1B—C2B—O1B	176.8 (3)
N1A—C1A—C2A—O2A	−163.1 (3)	N1B—C1B—C2B—O2B	172.9 (3)
C4A—C1A—C2A—O2A	13.0 (4)	C4B—C1B—C2B—O2B	−3.2 (4)
N1A—C1A—C4A—C9A	−145.6 (3)	N1B—C1B—C4B—C5B	−43.2 (4)
C2A—C1A—C4A—C9A	38.1 (5)	C2B—C1B—C4B—C5B	133.2 (3)
N1A—C1A—C4A—C5A	32.0 (5)	N1B—C1B—C4B—C9B	134.9 (3)
C2A—C1A—C4A—C5A	−144.4 (3)	C2B—C1B—C4B—C9B	−48.7 (5)
C9A—C4A—C5A—C6A	−2.1 (5)	C9B—C4B—C5B—C6B	1.3 (5)
C1A—C4A—C5A—C6A	−179.7 (3)	C1B—C4B—C5B—C6B	179.5 (3)
C4A—C5A—C6A—C7A	−0.5 (5)	C4B—C5B—C6B—C7B	−1.9 (5)
C5A—C6A—C7A—C8A	2.4 (5)	C5B—C6B—C7B—C8B	1.2 (5)
C5A—C6A—C7A—Br1A	−178.0 (3)	C5B—C6B—C7B—Br1B	−179.6 (2)
C6A—C7A—C8A—C9A	−1.6 (5)	C6B—C7B—C8B—C9B	0.1 (5)
Br1A—C7A—C8A—C9A	178.8 (3)	Br1B—C7B—C8B—C9B	−179.1 (2)
C7A—C8A—C9A—C4A	−1.1 (5)	C7B—C8B—C9B—C4B	−0.6 (5)
C5A—C4A—C9A—C8A	2.9 (5)	C5B—C4B—C9B—C8B	−0.1 (5)
C1A—C4A—C9A—C8A	−179.6 (3)	C1B—C4B—C9B—C8B	−178.2 (3)
C15A—C10A—C11A—C12A	0.3 (6)	C15B—C10B—C11B—C12B	0.9 (5)
N2A—C10A—C11A—C12A	−179.9 (3)	N2B—C10B—C11B—C12B	180.0 (3)
C10A—C11A—C12A—C13A	−0.2 (6)	C10B—C11B—C12B—C13B	−1.1 (5)
C10A—C11A—C12A—C16A	−179.8 (4)	C10B—C11B—C12B—C16B	179.1 (4)
C11A—C12A—C13A—C14A	0.5 (6)	C11B—C12B—C13B—C14B	0.9 (5)
C16A—C12A—C13A—C14A	−179.9 (4)	C16B—C12B—C13B—C14B	−179.3 (4)
C12A—C13A—C14A—C15A	−0.8 (5)	C12B—C13B—C14B—C15B	−0.4 (5)
C12A—C13A—C14A—C17A	179.1 (4)	C12B—C13B—C14B—C17B	178.7 (3)
C11A—C10A—C15A—C14A	−0.5 (5)	C11B—C10B—C15B—C14B	−0.4 (5)
N2A—C10A—C15A—C14A	179.6 (3)	N2B—C10B—C15B—C14B	−179.5 (3)
C13A—C14A—C15A—C10A	0.8 (5)	C13B—C14B—C15B—C10B	0.2 (5)
C17A—C14A—C15A—C10A	−179.1 (3)	C17B—C14B—C15B—C10B	−178.9 (3)
C2A—C1A—N1A—N2A	−4.7 (5)	C2B—C1B—N1B—N2B	0.9 (5)
C4A—C1A—N1A—N2A	179.0 (3)	C4B—C1B—N1B—N2B	177.2 (3)
C1A—N1A—N2A—C10A	−179.6 (3)	C1B—N1B—N2B—C10B	−177.8 (3)
C11A—C10A—N2A—N1A	178.1 (3)	C15B—C10B—N2B—N1B	−4.6 (5)
C15A—C10A—N2A—N1A	−2.0 (5)	C11B—C10B—N2B—N1B	176.3 (3)
O1A—C2A—O2A—C3A	0.4 (5)	O1B—C2B—O2B—C3B	2.0 (5)
C1A—C2A—O2A—C3A	176.7 (3)	C1B—C2B—O2B—C3B	−178.0 (3)

### Methyl (Z)-2-(4-bromophenyl)-2-[2-(3,5-dimethylphenyl)hydrazin-1-ylidene]acetate (2) Hydrogen-bond geometry (Å, º)

**Table:**

*D*—H···*A*	*D*—H	H···*A*	*D*···*A*	*D*—H···*A*
N2*A*—H2*A*···O1*A*	1.00 (5)	1.82 (5)	2.631 (4)	136 (4)
N2*B*—H2*B*···O1*B*	0.81 (5)	2.03 (5)	2.645 (4)	133 (5)
C9*A*—H9*A*···O2*A*	0.95	2.47	2.827 (4)	102
C9*B*—H9*B*···O2*B*	0.95	2.58	2.898 (4)	100
C5*A*—H5*A*···*Cg*3^i^	0.95	2.88	3.637 (4)	137

Symmetry code: (i) *x*−1, *y*, *z*.

### Methyl (Z)-2-[2-(4-methoxyphenyl)hydrazin-1-ylidene]-2-(3-nitrophenyl)acetate (3) Crystal data

**Table:**

C_16_H_15_N_3_O_5_	*F*(000) = 1376
*M**_r_* = 329.31	*D*_x_ = 1.434 Mg m^−^^3^
Monoclinic, *C*2/*c*	Cu *K*α radiation, λ = 1.54184 Å
*a* = 18.8022 (5) Å	Cell parameters from 4042 reflections
*b* = 21.9649 (6) Å	θ = 4.0–78.2°
*c* = 7.43092 (15) Å	µ = 0.91 mm^−^^1^
β = 96.156 (2)°	*T* = 100 K
*V* = 3051.19 (13) Å^3^	Prismatic needle, yellow
*Z* = 8	0.29 × 0.10 × 0.09 mm

### Methyl (Z)-2-[2-(4-methoxyphenyl)hydrazin-1-ylidene]-2-(3-nitrophenyl)acetate (3) Data collection

**Table:**

Rigaku XtaLAB Synergy-S, HyPix-6000HE area-detector diffractometer	2634 reflections with *I* > 2σ(*I*)
Radiation source: micro-focus sealed X-ray tube	*R*_int_ = 0.072
φ and ω scans	θ_max_ = 80.0°, θ_min_ = 3.1°
Absorption correction: gaussian (CrysAlisPro; Rigaku OD, 2021).	*h* = −23→23
*T*_min_ = 0.322, *T*_max_ = 1.000	*k* = −28→27
21181 measured reflections	*l* = −6→9
3303 independent reflections	

### Methyl (Z)-2-[2-(4-methoxyphenyl)hydrazin-1-ylidene]-2-(3-nitrophenyl)acetate (3) Refinement

**Table:**

Refinement on *F*^2^	Secondary atom site location: difference Fourier map
Least-squares matrix: full	Hydrogen site location: mixed
*R*[*F*^2^ > 2σ(*F*^2^)] = 0.046	H atoms treated by a mixture of independent and constrained refinement
*wR*(*F*^2^) = 0.126	*w* = 1/[σ^2^(*F*_o_^2^) + (0.0486*P*)^2^ + 2.64*P*] where *P* = (*F*_o_^2^ + 2*F*_c_^2^)/3
*S* = 1.07	(Δ/σ)_max_ = 0.001
3303 reflections	Δρ_max_ = 0.27 e Å^−^^3^
223 parameters	Δρ_min_ = −0.25 e Å^−^^3^
0 restraints	Extinction correction: SHELXL, Fc^*^=kFc[1+0.001xFc^2^λ^3^/sin(2θ)]^-1/4^
Primary atom site location: difference Fourier map	Extinction coefficient: 0.00022 (2)

### Methyl (Z)-2-[2-(4-methoxyphenyl)hydrazin-1-ylidene]-2-(3-nitrophenyl)acetate (3) Special details

**Table:**

Geometry. All esds (except the esd in the dihedral angle between two l.s. planes) are estimated using the full covariance matrix. The cell esds are taken into account individually in the estimation of esds in distances, angles and torsion angles; correlations between esds in cell parameters are only used when they are defined by crystal symmetry. An approximate (isotropic) treatment of cell esds is used for estimating esds involving l.s. planes.

### Methyl (Z)-2-[2-(4-methoxyphenyl)hydrazin-1-ylidene]-2-(3-nitrophenyl)acetate (3) Fractional atomic coordinates and isotropic or equivalent isotropic displacement parameters (Å^2^)

**Table:**

	*x*	*y*	*z*	*U*_iso_*/*U*_eq_	
O1	0.61696 (7)	0.61943 (6)	0.38295 (16)	0.0270 (3)	
O2	0.68370 (6)	0.70446 (6)	0.37126 (17)	0.0268 (3)	
O3	0.63758 (7)	0.86543 (6)	−0.02805 (16)	0.0303 (3)	
O4	0.59273 (7)	0.95144 (6)	0.04857 (17)	0.0288 (3)	
O5	0.24312 (6)	0.55365 (6)	0.76639 (16)	0.0246 (3)	
N1	0.50639 (8)	0.69827 (7)	0.50010 (18)	0.0221 (3)	
N2	0.49323 (8)	0.63946 (7)	0.5152 (2)	0.0243 (3)	
H2	0.5246 (12)	0.6133 (11)	0.488 (3)	0.029*	
N3	0.60948 (8)	0.89818 (7)	0.07922 (18)	0.0230 (3)	
C1	0.56661 (9)	0.71660 (8)	0.4425 (2)	0.0210 (3)	
C2	0.62303 (9)	0.67454 (8)	0.3949 (2)	0.0220 (4)	
C3	0.74375 (10)	0.66734 (9)	0.3324 (3)	0.0317 (4)	
H3A	0.7313	0.6450	0.2192	0.048*	
H3B	0.7554	0.6384	0.4314	0.048*	
H3C	0.7852	0.6935	0.3203	0.048*	
C4	0.57519 (8)	0.78340 (8)	0.4289 (2)	0.0197 (3)	
C5	0.59311 (8)	0.80907 (8)	0.2672 (2)	0.0202 (3)	
H5	0.6038	0.7840	0.1695	0.024*	
C6	0.59483 (9)	0.87167 (8)	0.2535 (2)	0.0200 (3)	
C7	0.58109 (9)	0.91057 (8)	0.3922 (2)	0.0225 (3)	
H7	0.5819	0.9535	0.3770	0.027*	
C8	0.56615 (9)	0.88470 (9)	0.5545 (2)	0.0237 (4)	
H8	0.5582	0.9100	0.6539	0.028*	
C9	0.56288 (9)	0.82174 (8)	0.5716 (2)	0.0222 (4)	
H9	0.5520	0.8046	0.6828	0.027*	
C10	0.42944 (9)	0.61927 (8)	0.5796 (2)	0.0228 (4)	
C11	0.42116 (9)	0.55701 (8)	0.6003 (2)	0.0240 (4)	
H11	0.4579	0.5302	0.5712	0.029*	
C12	0.35965 (9)	0.53308 (8)	0.6634 (2)	0.0233 (4)	
H12	0.3545	0.4904	0.6778	0.028*	
C13	0.30601 (9)	0.57256 (8)	0.7047 (2)	0.0216 (4)	
C14	0.31391 (9)	0.63514 (8)	0.6835 (2)	0.0233 (4)	
H14	0.2770	0.6619	0.7117	0.028*	
C15	0.37540 (9)	0.65865 (8)	0.6213 (2)	0.0239 (4)	
H15	0.3806	0.7014	0.6073	0.029*	
C16	0.23032 (9)	0.48938 (8)	0.7635 (2)	0.0254 (4)	
H16A	0.2302	0.4742	0.6395	0.038*	
H16B	0.1839	0.4811	0.8069	0.038*	
H16C	0.2681	0.4689	0.8423	0.038*	

### Methyl (Z)-2-[2-(4-methoxyphenyl)hydrazin-1-ylidene]-2-(3-nitrophenyl)acetate (3) Atomic displacement parameters (Å^2^)

**Table:**

	*U*^11^	*U*^22^	*U*^33^	*U*^12^	*U*^13^	*U*^23^
O1	0.0297 (7)	0.0239 (7)	0.0277 (6)	−0.0003 (5)	0.0043 (5)	−0.0002 (5)
O2	0.0191 (6)	0.0262 (7)	0.0357 (7)	0.0005 (5)	0.0057 (5)	0.0010 (5)
O3	0.0396 (7)	0.0329 (7)	0.0196 (6)	−0.0022 (6)	0.0088 (5)	−0.0013 (5)
O4	0.0310 (7)	0.0246 (7)	0.0305 (6)	−0.0009 (6)	0.0024 (5)	0.0085 (5)
O5	0.0221 (6)	0.0250 (6)	0.0278 (6)	−0.0020 (5)	0.0071 (5)	0.0003 (5)
N1	0.0231 (7)	0.0240 (7)	0.0189 (6)	−0.0025 (6)	0.0002 (5)	0.0034 (6)
N2	0.0242 (7)	0.0233 (8)	0.0256 (7)	−0.0026 (6)	0.0041 (6)	0.0028 (6)
N3	0.0229 (7)	0.0258 (8)	0.0201 (7)	−0.0039 (6)	0.0015 (5)	0.0028 (6)
C1	0.0212 (7)	0.0249 (9)	0.0165 (7)	−0.0010 (7)	0.0005 (6)	0.0026 (6)
C2	0.0226 (8)	0.0255 (9)	0.0176 (7)	−0.0024 (7)	0.0013 (6)	0.0015 (6)
C3	0.0231 (9)	0.0322 (10)	0.0405 (10)	0.0045 (8)	0.0059 (8)	−0.0002 (8)
C4	0.0169 (7)	0.0236 (9)	0.0182 (7)	−0.0012 (6)	−0.0005 (6)	0.0021 (6)
C5	0.0189 (7)	0.0256 (9)	0.0160 (7)	−0.0002 (7)	0.0018 (6)	−0.0014 (6)
C6	0.0184 (7)	0.0245 (9)	0.0168 (7)	−0.0023 (6)	0.0012 (6)	0.0023 (6)
C7	0.0208 (7)	0.0213 (8)	0.0252 (8)	−0.0010 (7)	0.0020 (6)	−0.0022 (7)
C8	0.0214 (8)	0.0299 (9)	0.0199 (8)	0.0008 (7)	0.0035 (6)	−0.0034 (7)
C9	0.0189 (7)	0.0303 (9)	0.0178 (7)	−0.0004 (7)	0.0032 (6)	0.0024 (7)
C10	0.0238 (8)	0.0267 (9)	0.0178 (7)	−0.0045 (7)	0.0005 (6)	0.0011 (7)
C11	0.0226 (8)	0.0241 (9)	0.0256 (8)	0.0004 (7)	0.0046 (6)	0.0000 (7)
C12	0.0247 (8)	0.0217 (9)	0.0235 (8)	−0.0019 (7)	0.0029 (6)	0.0005 (7)
C13	0.0219 (8)	0.0261 (9)	0.0168 (7)	−0.0038 (7)	0.0018 (6)	0.0005 (6)
C14	0.0240 (8)	0.0253 (9)	0.0206 (8)	0.0007 (7)	0.0019 (6)	−0.0015 (7)
C15	0.0267 (8)	0.0229 (9)	0.0216 (8)	−0.0016 (7)	0.0006 (6)	0.0014 (7)
C16	0.0255 (8)	0.0262 (9)	0.0248 (8)	−0.0041 (7)	0.0040 (7)	0.0034 (7)

### Methyl (Z)-2-[2-(4-methoxyphenyl)hydrazin-1-ylidene]-2-(3-nitrophenyl)acetate (3) Geometric parameters (Å, º)

**Table:**

O1—C2	1.218 (2)	C5—H5	0.9500
O2—C2	1.344 (2)	C6—C7	1.385 (2)
O2—C3	1.447 (2)	C7—C8	1.388 (2)
O3—N3	1.2339 (19)	C7—H7	0.9500
O4—N3	1.227 (2)	C8—C9	1.391 (3)
O5—C13	1.3774 (19)	C8—H8	0.9500
O5—C16	1.432 (2)	C9—H9	0.9500
N1—C1	1.316 (2)	C10—C11	1.387 (2)
N1—N2	1.322 (2)	C10—C15	1.394 (3)
N2—C10	1.410 (2)	C11—C12	1.396 (2)
N2—H2	0.86 (2)	C11—H11	0.9500
N3—C6	1.472 (2)	C12—C13	1.389 (2)
C1—C2	1.478 (2)	C12—H12	0.9500
C1—C4	1.481 (2)	C13—C14	1.393 (2)
C3—H3A	0.9800	C14—C15	1.390 (2)
C3—H3B	0.9800	C14—H14	0.9500
C3—H3C	0.9800	C15—H15	0.9500
C4—C9	1.393 (2)	C16—H16A	0.9800
C4—C5	1.401 (2)	C16—H16B	0.9800
C5—C6	1.379 (2)	C16—H16C	0.9800
			
C2—O2—C3	116.24 (15)	C8—C7—H7	121.1
C13—O5—C16	116.19 (13)	C7—C8—C9	120.11 (15)
C1—N1—N2	120.14 (15)	C7—C8—H8	119.9
N1—N2—C10	120.66 (15)	C9—C8—H8	119.9
N1—N2—H2	119.4 (15)	C8—C9—C4	121.25 (15)
C10—N2—H2	119.9 (15)	C8—C9—H9	119.4
O4—N3—O3	123.74 (14)	C4—C9—H9	119.4
O4—N3—C6	118.16 (14)	C11—C10—C15	119.60 (16)
O3—N3—C6	118.10 (14)	C11—C10—N2	117.23 (16)
N1—C1—C2	123.46 (16)	C15—C10—N2	123.17 (17)
N1—C1—C4	115.42 (15)	C10—C11—C12	121.02 (16)
C2—C1—C4	121.12 (14)	C10—C11—H11	119.5
O1—C2—O2	123.38 (16)	C12—C11—H11	119.5
O1—C2—C1	125.07 (15)	C13—C12—C11	119.10 (16)
O2—C2—C1	111.52 (15)	C13—C12—H12	120.5
O2—C3—H3A	109.5	C11—C12—H12	120.5
O2—C3—H3B	109.5	O5—C13—C12	123.69 (16)
H3A—C3—H3B	109.5	O5—C13—C14	116.16 (15)
O2—C3—H3C	109.5	C12—C13—C14	120.14 (15)
H3A—C3—H3C	109.5	C15—C14—C13	120.44 (16)
H3B—C3—H3C	109.5	C15—C14—H14	119.8
C9—C4—C5	118.99 (16)	C13—C14—H14	119.8
C9—C4—C1	121.26 (14)	C14—C15—C10	119.70 (17)
C5—C4—C1	119.69 (15)	C14—C15—H15	120.1
C6—C5—C4	118.33 (15)	C10—C15—H15	120.1
C6—C5—H5	120.8	O5—C16—H16A	109.5
C4—C5—H5	120.8	O5—C16—H16B	109.5
C5—C6—C7	123.51 (15)	H16A—C16—H16B	109.5
C5—C6—N3	117.87 (14)	O5—C16—H16C	109.5
C7—C6—N3	118.58 (15)	H16A—C16—H16C	109.5
C6—C7—C8	117.73 (16)	H16B—C16—H16C	109.5
C6—C7—H7	121.1		
			
C1—N1—N2—C10	178.96 (15)	C5—C6—C7—C8	1.2 (3)
N2—N1—C1—C2	−1.1 (2)	N3—C6—C7—C8	178.90 (14)
N2—N1—C1—C4	179.31 (14)	C6—C7—C8—C9	−2.2 (2)
C3—O2—C2—O1	−0.9 (2)	C7—C8—C9—C4	0.8 (2)
C3—O2—C2—C1	177.22 (14)	C5—C4—C9—C8	1.8 (2)
N1—C1—C2—O1	9.1 (3)	C1—C4—C9—C8	−175.54 (15)
C4—C1—C2—O1	−171.35 (15)	N1—N2—C10—C11	−176.81 (14)
N1—C1—C2—O2	−169.00 (15)	N1—N2—C10—C15	3.5 (2)
C4—C1—C2—O2	10.5 (2)	C15—C10—C11—C12	−0.4 (3)
N1—C1—C4—C9	49.2 (2)	N2—C10—C11—C12	179.97 (15)
C2—C1—C4—C9	−130.37 (17)	C10—C11—C12—C13	0.3 (3)
N1—C1—C4—C5	−128.10 (16)	C16—O5—C13—C12	−8.3 (2)
C2—C1—C4—C5	52.3 (2)	C16—O5—C13—C14	171.31 (14)
C9—C4—C5—C6	−2.7 (2)	C11—C12—C13—O5	179.49 (15)
C1—C4—C5—C6	174.63 (14)	C11—C12—C13—C14	−0.1 (2)
C4—C5—C6—C7	1.3 (2)	O5—C13—C14—C15	−179.76 (14)
C4—C5—C6—N3	−176.41 (14)	C12—C13—C14—C15	−0.1 (2)
O4—N3—C6—C5	160.87 (15)	C13—C14—C15—C10	0.1 (2)
O3—N3—C6—C5	−18.7 (2)	C11—C10—C15—C14	0.1 (2)
O4—N3—C6—C7	−16.9 (2)	N2—C10—C15—C14	179.77 (15)
O3—N3—C6—C7	163.47 (15)		

### Methyl (Z)-2-[2-(4-methoxyphenyl)hydrazin-1-ylidene]-2-(3-nitrophenyl)acetate (3) Hydrogen-bond geometry (Å, º)

**Table:**

*D*—H···*A*	*D*—H	H···*A*	*D*···*A*	*D*—H···*A*
N2—H2···O1	0.86 (2)	1.98 (2)	2.657 (2)	134 (2)
C7—H7···O4^i^	0.95	2.44	3.245 (2)	142
C12—H12···O1^ii^	0.95	2.52	3.401 (2)	154

Symmetry codes: (i) *x*, −*y*+2, *z*+1/2; (ii) −*x*+1, −*y*+1, −*z*+1.

### Methyl (E)-2-(4-chlorophenyl)-2-(2-phenylhydrazin-1-ylidene)acetate (4) Crystal data

**Table:**

C_15_H_13_ClN_2_O_2_	*D*_x_ = 1.428 Mg m^−^^3^
*M**_r_* = 288.72	Cu *K*α radiation, λ = 1.54184 Å
Orthorhombic, *P**b**c**a*	Cell parameters from 35974 reflections
*a* = 15.86326 (8) Å	θ = 4.6–79.5°
*b* = 8.79608 (3) Å	µ = 2.55 mm^−^^1^
*c* = 19.24680 (8) Å	*T* = 100 K
*V* = 2685.59 (2) Å^3^	Prism, yellow
*Z* = 8	0.12 × 0.11 × 0.06 mm
*F*(000) = 1200	

### Methyl (E)-2-(4-chlorophenyl)-2-(2-phenylhydrazin-1-ylidene)acetate (4) Data collection

**Table:**

Rigaku XtaLAB Synergy-S, HyPix-6000HE area-detector diffractometer	2871 reflections with *I* > 2σ(*I*)
Radiation source: micro-focus sealed X-ray tube	*R*_int_ = 0.030
φ and ω scans	θ_max_ = 80.1°, θ_min_ = 4.6°
Absorption correction: multi-scan (CrysAlisPro; Rigaku OD, 2021).	*h* = −20→20
*T*_min_ = 0.676, *T*_max_ = 1.000	*k* = −11→10
51407 measured reflections	*l* = −24→24
2935 independent reflections	

### Methyl (E)-2-(4-chlorophenyl)-2-(2-phenylhydrazin-1-ylidene)acetate (4) Refinement

**Table:**

Refinement on *F*^2^	Primary atom site location: difference Fourier map
Least-squares matrix: full	Secondary atom site location: difference Fourier map
*R*[*F*^2^ > 2σ(*F*^2^)] = 0.030	Hydrogen site location: mixed
*wR*(*F*^2^) = 0.082	H atoms treated by a mixture of independent and constrained refinement
*S* = 1.08	*w* = 1/[σ^2^(*F*_o_^2^) + (0.0407*P*)^2^ + 1.4066*P*] where *P* = (*F*_o_^2^ + 2*F*_c_^2^)/3
2935 reflections	(Δ/σ)_max_ = 0.001
186 parameters	Δρ_max_ = 0.29 e Å^−^^3^
0 restraints	Δρ_min_ = −0.28 e Å^−^^3^

### Methyl (E)-2-(4-chlorophenyl)-2-(2-phenylhydrazin-1-ylidene)acetate (4) Special details

**Table:**

Geometry. All esds (except the esd in the dihedral angle between two l.s. planes) are estimated using the full covariance matrix. The cell esds are taken into account individually in the estimation of esds in distances, angles and torsion angles; correlations between esds in cell parameters are only used when they are defined by crystal symmetry. An approximate (isotropic) treatment of cell esds is used for estimating esds involving l.s. planes.

### Methyl (E)-2-(4-chlorophenyl)-2-(2-phenylhydrazin-1-ylidene)acetate (4) Fractional atomic coordinates and isotropic or equivalent isotropic displacement parameters (Å^2^)

**Table:**

	*x*	*y*	*z*	*U*_iso_*/*U*_eq_	
Cl1	0.62856 (2)	0.69084 (3)	0.30196 (2)	0.02208 (10)	
O1	0.55614 (6)	−0.13395 (10)	0.46392 (4)	0.02205 (19)	
O2	0.52650 (5)	0.03451 (9)	0.37933 (4)	0.01828 (18)	
N1	0.68620 (6)	0.05063 (11)	0.50371 (5)	0.0165 (2)	
N2	0.74780 (6)	0.14132 (11)	0.52647 (5)	0.0176 (2)	
H2	0.7585 (10)	0.227 (2)	0.5070 (9)	0.025 (4)*	
C1	0.63703 (7)	0.09638 (13)	0.45406 (6)	0.0161 (2)	
C2	0.57050 (7)	−0.01517 (13)	0.43440 (6)	0.0168 (2)	
C3	0.45741 (7)	−0.06150 (15)	0.35765 (6)	0.0213 (2)	
H3A	0.4278	−0.0137	0.3187	0.032*	
H3B	0.4182	−0.0753	0.3965	0.032*	
H3C	0.4794	−0.1607	0.3431	0.032*	
C4	0.64041 (7)	0.24740 (13)	0.41905 (6)	0.0157 (2)	
C5	0.61040 (8)	0.37765 (14)	0.45216 (6)	0.0188 (2)	
H5	0.5916	0.3719	0.4989	0.023*	
C6	0.60782 (8)	0.51597 (14)	0.41715 (6)	0.0194 (2)	
H6	0.5869	0.6046	0.4394	0.023*	
C7	0.63640 (7)	0.52207 (13)	0.34913 (6)	0.0176 (2)	
C8	0.66907 (8)	0.39514 (14)	0.31583 (6)	0.0197 (2)	
H8	0.6903	0.4024	0.2698	0.024*	
C9	0.67013 (7)	0.25752 (13)	0.35102 (6)	0.0188 (2)	
H9	0.6913	0.1693	0.3285	0.023*	
C10	0.79852 (7)	0.09200 (13)	0.58191 (6)	0.0155 (2)	
C11	0.77436 (7)	−0.03165 (13)	0.62277 (6)	0.0167 (2)	
H11	0.7228	−0.0829	0.6137	0.020*	
C12	0.82608 (8)	−0.07924 (13)	0.67669 (6)	0.0196 (2)	
H12	0.8101	−0.1646	0.7039	0.023*	
C13	0.90101 (8)	−0.00373 (14)	0.69152 (6)	0.0203 (2)	
H13	0.9360	−0.0367	0.7286	0.024*	
C14	0.92395 (7)	0.12079 (13)	0.65114 (6)	0.0187 (2)	
H14	0.9746	0.1740	0.6613	0.022*	
C15	0.87377 (7)	0.16839 (13)	0.59610 (6)	0.0169 (2)	
H15	0.8905	0.2523	0.5683	0.020*	

### Methyl (E)-2-(4-chlorophenyl)-2-(2-phenylhydrazin-1-ylidene)acetate (4) Atomic displacement parameters (Å^2^)

**Table:**

	*U*^11^	*U*^22^	*U*^33^	*U*^12^	*U*^13^	*U*^23^
Cl1	0.02274 (16)	0.01621 (15)	0.02730 (17)	0.00088 (10)	−0.00065 (10)	0.00687 (10)
O1	0.0299 (5)	0.0155 (4)	0.0207 (4)	−0.0003 (3)	−0.0038 (3)	0.0027 (3)
O2	0.0199 (4)	0.0184 (4)	0.0165 (4)	−0.0008 (3)	−0.0041 (3)	0.0022 (3)
N1	0.0190 (5)	0.0163 (4)	0.0143 (4)	0.0019 (4)	−0.0007 (3)	−0.0008 (4)
N2	0.0214 (5)	0.0146 (4)	0.0168 (4)	−0.0008 (4)	−0.0033 (4)	0.0031 (4)
C1	0.0200 (5)	0.0146 (5)	0.0138 (5)	0.0031 (4)	−0.0002 (4)	−0.0006 (4)
C2	0.0209 (5)	0.0151 (5)	0.0143 (5)	0.0043 (4)	−0.0002 (4)	−0.0010 (4)
C3	0.0182 (5)	0.0247 (6)	0.0211 (5)	−0.0022 (5)	−0.0020 (4)	−0.0009 (5)
C4	0.0165 (5)	0.0148 (5)	0.0159 (5)	0.0010 (4)	−0.0042 (4)	0.0007 (4)
C5	0.0241 (6)	0.0176 (6)	0.0146 (5)	0.0012 (5)	−0.0016 (4)	−0.0013 (4)
C6	0.0226 (6)	0.0152 (5)	0.0204 (6)	0.0022 (4)	−0.0028 (4)	−0.0021 (4)
C7	0.0162 (5)	0.0149 (5)	0.0216 (6)	−0.0013 (4)	−0.0039 (4)	0.0035 (4)
C8	0.0206 (6)	0.0200 (6)	0.0184 (5)	0.0016 (4)	0.0017 (4)	0.0025 (4)
C9	0.0209 (5)	0.0168 (5)	0.0187 (5)	0.0028 (4)	0.0008 (4)	−0.0005 (4)
C10	0.0181 (5)	0.0151 (5)	0.0134 (5)	0.0040 (4)	0.0002 (4)	−0.0010 (4)
C11	0.0183 (5)	0.0147 (5)	0.0172 (5)	−0.0009 (4)	−0.0005 (4)	−0.0003 (4)
C12	0.0256 (6)	0.0167 (5)	0.0164 (5)	0.0004 (4)	−0.0005 (4)	0.0030 (4)
C13	0.0220 (6)	0.0218 (6)	0.0173 (5)	0.0042 (5)	−0.0037 (4)	0.0005 (4)
C14	0.0170 (5)	0.0194 (5)	0.0197 (5)	0.0006 (4)	−0.0001 (4)	−0.0036 (4)
C15	0.0183 (5)	0.0149 (5)	0.0176 (5)	0.0006 (4)	0.0031 (4)	−0.0005 (4)

### Methyl (E)-2-(4-chlorophenyl)-2-(2-phenylhydrazin-1-ylidene)acetate (4) Geometric parameters (Å, º)

**Table:**

Cl1—C7	1.7446 (12)	C6—C7	1.3864 (17)
O1—C2	1.2109 (14)	C6—H6	0.9500
O2—C2	1.3423 (14)	C7—C8	1.3878 (17)
O2—C3	1.4451 (14)	C8—C9	1.3872 (16)
N1—C1	1.2975 (15)	C8—H8	0.9500
N1—N2	1.3354 (14)	C9—H9	0.9500
N2—C10	1.4050 (14)	C10—C11	1.3959 (16)
N2—H2	0.861 (18)	C10—C15	1.3967 (16)
C1—C2	1.4899 (16)	C11—C12	1.3876 (16)
C1—C4	1.4905 (16)	C11—H11	0.9500
C3—H3A	0.9800	C12—C13	1.3911 (18)
C3—H3B	0.9800	C12—H12	0.9500
C3—H3C	0.9800	C13—C14	1.3914 (17)
C4—C9	1.3945 (16)	C13—H13	0.9500
C4—C5	1.3948 (16)	C14—C15	1.3897 (17)
C5—C6	1.3914 (17)	C14—H14	0.9500
C5—H5	0.9500	C15—H15	0.9500
			
C2—O2—C3	115.60 (9)	C6—C7—Cl1	120.07 (9)
C1—N1—N2	119.75 (10)	C8—C7—Cl1	118.09 (9)
N1—N2—C10	118.94 (9)	C9—C8—C7	118.75 (11)
N1—N2—H2	121.8 (11)	C9—C8—H8	120.6
C10—N2—H2	119.2 (11)	C7—C8—H8	120.6
N1—C1—C2	114.11 (10)	C8—C9—C4	120.66 (11)
N1—C1—C4	126.00 (11)	C8—C9—H9	119.7
C2—C1—C4	119.84 (10)	C4—C9—H9	119.7
O1—C2—O2	123.61 (11)	C11—C10—C15	119.96 (10)
O1—C2—C1	125.62 (11)	C11—C10—N2	120.74 (10)
O2—C2—C1	110.76 (10)	C15—C10—N2	119.30 (10)
O2—C3—H3A	109.5	C12—C11—C10	119.61 (11)
O2—C3—H3B	109.5	C12—C11—H11	120.2
H3A—C3—H3B	109.5	C10—C11—H11	120.2
O2—C3—H3C	109.5	C11—C12—C13	120.97 (11)
H3A—C3—H3C	109.5	C11—C12—H12	119.5
H3B—C3—H3C	109.5	C13—C12—H12	119.5
C9—C4—C5	119.48 (11)	C12—C13—C14	119.00 (11)
C9—C4—C1	119.57 (10)	C12—C13—H13	120.5
C5—C4—C1	120.88 (10)	C14—C13—H13	120.5
C6—C5—C4	120.46 (11)	C15—C14—C13	120.87 (11)
C6—C5—H5	119.8	C15—C14—H14	119.6
C4—C5—H5	119.8	C13—C14—H14	119.6
C7—C6—C5	118.79 (11)	C14—C15—C10	119.58 (11)
C7—C6—H6	120.6	C14—C15—H15	120.2
C5—C6—H6	120.6	C10—C15—H15	120.2
C6—C7—C8	121.81 (11)		
			
C1—N1—N2—C10	177.79 (10)	C5—C6—C7—Cl1	176.33 (9)
N2—N1—C1—C2	−179.28 (9)	C6—C7—C8—C9	2.39 (18)
N2—N1—C1—C4	−1.64 (17)	Cl1—C7—C8—C9	−175.45 (9)
C3—O2—C2—O1	1.85 (16)	C7—C8—C9—C4	−1.20 (18)
C3—O2—C2—C1	−177.15 (9)	C5—C4—C9—C8	−0.84 (17)
N1—C1—C2—O1	5.92 (17)	C1—C4—C9—C8	176.03 (11)
C4—C1—C2—O1	−171.89 (11)	N1—N2—C10—C11	−14.31 (16)
N1—C1—C2—O2	−175.10 (9)	N1—N2—C10—C15	165.87 (10)
C4—C1—C2—O2	7.09 (14)	C15—C10—C11—C12	−0.95 (17)
N1—C1—C4—C9	108.05 (14)	N2—C10—C11—C12	179.23 (10)
C2—C1—C4—C9	−74.43 (14)	C10—C11—C12—C13	1.22 (18)
N1—C1—C4—C5	−75.13 (16)	C11—C12—C13—C14	−0.27 (18)
C2—C1—C4—C5	102.39 (13)	C12—C13—C14—C15	−0.95 (18)
C9—C4—C5—C6	1.78 (18)	C13—C14—C15—C10	1.21 (17)
C1—C4—C5—C6	−175.04 (11)	C11—C10—C15—C14	−0.25 (17)
C4—C5—C6—C7	−0.65 (18)	N2—C10—C15—C14	179.58 (10)
C5—C6—C7—C8	−1.47 (18)		

### Methyl (E)-2-(4-chlorophenyl)-2-(2-phenylhydrazin-1-ylidene)acetate (4) Hydrogen-bond geometry (Å, º)

**Table:**

*D*—H···*A*	*D*—H	H···*A*	*D*···*A*	*D*—H···*A*
C3—H3*B*···N1^i^	0.98	2.55	3.5099 (15)	168
C3—H3*C*···Cl1^ii^	0.98	2.82	3.6422 (12)	142
C6—H6···O1^iii^	0.95	2.40	3.3113 (15)	161
C15—H15···O1^iv^	0.95	2.40	3.2759 (14)	153
C14—H14···*Cg*1^v^	0	2.80	3.4792 (12)	129

Symmetry codes: (i) −*x*+1, −*y*, −*z*+1; (ii) *x*, *y*−1, *z*; (iii) *x*, *y*+1, *z*; (iv) −*x*+3/2, *y*+1/2, *z*; (v) −*x*, *y*+1/2, −*z*+3/2.

### Methyl (Z)-2-[2-(4-bromophenyl)hydrazin-1-ylidene]-2-(4-chlorophenyl)acetate (5) Crystal data

**Table:**

C_15_H_12_BrClN_2_O_2_	*D*_x_ = 1.628 Mg m^−^^3^
*M**_r_* = 367.62	Synchrotron radiation, λ = 0.75270 Å
Orthorhombic, *P**c**a*2_1_	Cell parameters from 1000 reflections
*a* = 14.0199 (16) Å	θ = 2.0–30.0°
*b* = 16.5940 (19) Å	µ = 3.35 mm^−^^1^
*c* = 6.4471 (9) Å	*T* = 100 K
*V* = 1499.9 (3) Å^3^	Prism, yellow
*Z* = 4	0.18 × 0.15 × 0.13 mm
*F*(000) = 736	

### Methyl (Z)-2-[2-(4-bromophenyl)hydrazin-1-ylidene]-2-(4-chlorophenyl)acetate (5) Data collection

**Table:**

Rayonix SX165 CCD diffractometer	3677 reflections with *I* > 2σ(*I*)
/f scan	*R*_int_ = 0.039
Absorption correction: multi-scan (Scala; Evans, 2006)	θ_max_ = 30.9°, θ_min_ = 2.0°
*T*_min_ = 0.514, *T*_max_ = 0.633	*h* = −19→18
12123 measured reflections	*k* = −18→22
3908 independent reflections	*l* = −8→8

### Methyl (Z)-2-[2-(4-bromophenyl)hydrazin-1-ylidene]-2-(4-chlorophenyl)acetate (5) Refinement

**Table:**

Refinement on *F*^2^	Hydrogen site location: mixed
Least-squares matrix: full	H atoms treated by a mixture of independent and constrained refinement
*R*[*F*^2^ > 2σ(*F*^2^)] = 0.040	*w* = 1/[σ^2^(*F*_o_^2^) + (0.0506*P*)^2^ + 2.3974*P*] where *P* = (*F*_o_^2^ + 2*F*_c_^2^)/3
*wR*(*F*^2^) = 0.110	(Δ/σ)_max_ = 0.001
*S* = 1.09	Δρ_max_ = 1.27 e Å^−^^3^
3908 reflections	Δρ_min_ = −0.67 e Å^−^^3^
196 parameters	Extinction correction: SHELXL, Fc^*^=kFc[1+0.001xFc^2^λ^3^/sin(2θ)]^-1/4^
1 restraint	Extinction coefficient: 0.014 (1)
Primary atom site location: difference Fourier map	Absolute structure: Refined as an inversion twin.
Secondary atom site location: difference Fourier map	Absolute structure parameter: 0.167 (15)

### Methyl (Z)-2-[2-(4-bromophenyl)hydrazin-1-ylidene]-2-(4-chlorophenyl)acetate (5) Special details

**Table:**

Geometry. All esds (except the esd in the dihedral angle between two l.s. planes) are estimated using the full covariance matrix. The cell esds are taken into account individually in the estimation of esds in distances, angles and torsion angles; correlations between esds in cell parameters are only used when they are defined by crystal symmetry. An approximate (isotropic) treatment of cell esds is used for estimating esds involving l.s. planes.
Refinement. Refined as a 2-component inversion twin.

### Methyl (Z)-2-[2-(4-bromophenyl)hydrazin-1-ylidene]-2-(4-chlorophenyl)acetate (5) Fractional atomic coordinates and isotropic or equivalent isotropic displacement parameters (Å^2^)

**Table:**

	*x*	*y*	*z*	*U*_iso_*/*U*_eq_	
Br1	0.35303 (3)	0.07176 (3)	0.51060 (10)	0.03049 (18)	
Cl1	0.86147 (9)	0.63637 (7)	0.4619 (3)	0.0430 (5)	
O1	0.95706 (19)	0.14905 (16)	0.5120 (8)	0.0231 (5)	
O2	1.04385 (18)	0.26332 (17)	0.5082 (9)	0.0251 (6)	
N1	0.7900 (2)	0.24954 (19)	0.5158 (9)	0.0196 (6)	
N2	0.7689 (2)	0.17193 (19)	0.5187 (9)	0.0203 (6)	
H2	0.816 (4)	0.133 (3)	0.534 (11)	0.028 (14)*	
C1	0.8774 (3)	0.2762 (2)	0.5109 (10)	0.0192 (7)	
C2	0.9619 (3)	0.2222 (2)	0.5112 (11)	0.0204 (6)	
C3	1.1298 (3)	0.2153 (3)	0.5058 (17)	0.0320 (9)	
H3A	1.1856	0.2509	0.5035	0.048*	
H3B	1.1302	0.1810	0.3821	0.048*	
H3C	1.1320	0.1815	0.6303	0.048*	
C4	0.8846 (3)	0.3657 (2)	0.5107 (10)	0.0193 (6)	
C5	0.8273 (4)	0.4089 (3)	0.6459 (8)	0.0273 (9)	
H5	0.7915	0.3809	0.7482	0.033*	
C6	0.8212 (4)	0.4922 (3)	0.6349 (9)	0.0306 (10)	
H6	0.7813	0.5212	0.7278	0.037*	
C7	0.8734 (3)	0.5318 (3)	0.4883 (12)	0.0283 (11)	
C8	0.9341 (4)	0.4911 (3)	0.3510 (8)	0.0296 (10)	
H8	0.9705	0.5197	0.2508	0.035*	
C9	0.9397 (4)	0.4077 (3)	0.3655 (8)	0.0271 (9)	
H9	0.9813	0.3788	0.2760	0.033*	
C10	0.6727 (3)	0.1497 (2)	0.5157 (11)	0.0197 (6)	
C11	0.6488 (3)	0.0683 (2)	0.5221 (18)	0.0257 (9)	
H11	0.6978	0.0289	0.5285	0.031*	
C12	0.5535 (3)	0.0441 (2)	0.5193 (12)	0.0260 (8)	
H12	0.5370	−0.0115	0.5219	0.031*	
C13	0.4838 (2)	0.1026 (2)	0.5125 (12)	0.0220 (7)	
C14	0.5058 (3)	0.1841 (2)	0.5129 (11)	0.0214 (7)	
H14	0.4562	0.2231	0.5133	0.026*	
C15	0.6005 (3)	0.2082 (2)	0.5128 (11)	0.0198 (7)	
H15	0.6164	0.2639	0.5109	0.024*	

### Methyl (Z)-2-[2-(4-bromophenyl)hydrazin-1-ylidene]-2-(4-chlorophenyl)acetate (5) Atomic displacement parameters (Å^2^)

**Table:**

	*U*^11^	*U*^22^	*U*^33^	*U*^12^	*U*^13^	*U*^23^
Br1	0.0175 (2)	0.0280 (2)	0.0460 (3)	−0.00531 (13)	−0.0003 (3)	−0.0011 (3)
Cl1	0.0338 (6)	0.0200 (5)	0.0750 (14)	−0.0015 (4)	−0.0054 (6)	0.0041 (6)
O1	0.0214 (12)	0.0224 (12)	0.0255 (13)	0.0014 (10)	−0.0026 (19)	−0.0035 (19)
O2	0.0157 (11)	0.0254 (13)	0.0343 (14)	−0.0009 (10)	−0.001 (2)	−0.001 (2)
N1	0.0205 (14)	0.0213 (14)	0.0168 (13)	−0.0035 (11)	0.002 (2)	−0.0016 (18)
N2	0.0156 (13)	0.0185 (13)	0.0268 (15)	0.0000 (10)	0.000 (2)	0.000 (2)
C1	0.0175 (15)	0.0205 (15)	0.0195 (15)	−0.0019 (12)	−0.010 (3)	−0.003 (2)
C2	0.0183 (15)	0.0248 (16)	0.0183 (15)	−0.0013 (12)	0.000 (3)	−0.001 (2)
C3	0.0186 (17)	0.033 (2)	0.044 (2)	0.0030 (15)	−0.009 (4)	−0.003 (3)
C4	0.0165 (14)	0.0210 (15)	0.0205 (15)	−0.0029 (12)	0.001 (2)	0.001 (3)
C5	0.023 (2)	0.027 (2)	0.031 (2)	−0.0007 (19)	0.0050 (18)	−0.0013 (18)
C6	0.023 (2)	0.027 (2)	0.042 (3)	−0.0032 (18)	0.006 (2)	0.000 (2)
C7	0.0209 (17)	0.0198 (17)	0.044 (3)	−0.0005 (14)	−0.002 (3)	0.007 (2)
C8	0.024 (2)	0.034 (3)	0.030 (2)	−0.0049 (19)	0.0017 (19)	0.0018 (19)
C9	0.023 (2)	0.034 (2)	0.025 (2)	−0.0040 (19)	0.0067 (18)	0.0016 (19)
C10	0.0166 (15)	0.0205 (15)	0.0218 (16)	−0.0020 (12)	−0.004 (2)	−0.002 (2)
C11	0.0201 (18)	0.0171 (16)	0.040 (3)	0.0017 (13)	0.001 (3)	0.000 (2)
C12	0.0218 (17)	0.0168 (15)	0.040 (2)	−0.0019 (13)	−0.004 (3)	0.003 (3)
C13	0.0150 (14)	0.0218 (16)	0.0292 (17)	−0.0024 (12)	0.000 (3)	0.000 (2)
C14	0.0198 (15)	0.0183 (15)	0.0262 (16)	0.0013 (12)	0.000 (3)	0.000 (2)
C15	0.0209 (16)	0.0185 (15)	0.0202 (15)	−0.0010 (12)	−0.001 (3)	−0.002 (2)

### Methyl (Z)-2-[2-(4-bromophenyl)hydrazin-1-ylidene]-2-(4-chlorophenyl)acetate (5) Geometric parameters (Å, º)

**Table:**

Br1—C13	1.904 (3)	C5—H5	0.9500
Cl1—C7	1.752 (4)	C6—C7	1.365 (8)
O1—C2	1.217 (4)	C6—H6	0.9500
O2—C2	1.335 (4)	C7—C8	1.401 (8)
O2—C3	1.445 (5)	C8—C9	1.390 (7)
N1—C1	1.303 (5)	C8—H8	0.9500
N1—N2	1.322 (4)	C9—H9	0.9500
N2—C10	1.397 (5)	C10—C11	1.392 (5)
N2—H2	0.93 (6)	C10—C15	1.403 (5)
C1—C2	1.485 (5)	C11—C12	1.396 (5)
C1—C4	1.489 (5)	C11—H11	0.9500
C3—H3A	0.9800	C12—C13	1.379 (5)
C3—H3B	0.9800	C12—H12	0.9500
C3—H3C	0.9800	C13—C14	1.385 (5)
C4—C5	1.385 (7)	C14—C15	1.388 (5)
C4—C9	1.399 (7)	C14—H14	0.9500
C5—C6	1.387 (7)	C15—H15	0.9500
			
C2—O2—C3	115.9 (3)	C6—C7—Cl1	119.5 (4)
C1—N1—N2	122.8 (3)	C8—C7—Cl1	118.3 (5)
N1—N2—C10	118.2 (3)	C9—C8—C7	118.1 (5)
N1—N2—H2	121 (3)	C9—C8—H8	120.9
C10—N2—H2	121 (3)	C7—C8—H8	120.9
N1—C1—C2	123.1 (3)	C8—C9—C4	120.6 (5)
N1—C1—C4	113.7 (3)	C8—C9—H9	119.7
C2—C1—C4	123.2 (3)	C4—C9—H9	119.7
O1—C2—O2	123.9 (3)	C11—C10—N2	119.2 (3)
O1—C2—C1	123.8 (3)	C11—C10—C15	119.8 (3)
O2—C2—C1	112.2 (3)	N2—C10—C15	120.9 (3)
O2—C3—H3A	109.5	C10—C11—C12	120.7 (3)
O2—C3—H3B	109.5	C10—C11—H11	119.7
H3A—C3—H3B	109.5	C12—C11—H11	119.7
O2—C3—H3C	109.5	C13—C12—C11	118.4 (3)
H3A—C3—H3C	109.5	C13—C12—H12	120.8
H3B—C3—H3C	109.5	C11—C12—H12	120.8
C5—C4—C9	119.0 (4)	C12—C13—C14	122.0 (3)
C5—C4—C1	118.5 (5)	C12—C13—Br1	119.5 (3)
C9—C4—C1	122.3 (5)	C14—C13—Br1	118.4 (3)
C4—C5—C6	121.3 (5)	C13—C14—C15	119.6 (3)
C4—C5—H5	119.4	C13—C14—H14	120.2
C6—C5—H5	119.4	C15—C14—H14	120.2
C7—C6—C5	118.8 (5)	C14—C15—C10	119.4 (3)
C7—C6—H6	120.6	C14—C15—H15	120.3
C5—C6—H6	120.6	C10—C15—H15	120.3
C6—C7—C8	122.1 (4)		
			
C1—N1—N2—C10	−177.5 (6)	C6—C7—C8—C9	0.7 (9)
N2—N1—C1—C2	−0.9 (11)	Cl1—C7—C8—C9	−176.8 (4)
N2—N1—C1—C4	−179.3 (6)	C7—C8—C9—C4	1.2 (8)
C3—O2—C2—O1	0.0 (11)	C5—C4—C9—C8	−2.7 (8)
C3—O2—C2—C1	−179.3 (7)	C1—C4—C9—C8	170.7 (5)
N1—C1—C2—O1	1.5 (11)	N1—N2—C10—C11	−179.0 (8)
C4—C1—C2—O1	179.8 (7)	N1—N2—C10—C15	−1.0 (10)
N1—C1—C2—O2	−179.2 (6)	N2—C10—C11—C12	180.0 (8)
C4—C1—C2—O2	−0.9 (9)	C15—C10—C11—C12	2.0 (14)
N1—C1—C4—C5	44.2 (8)	C10—C11—C12—C13	−0.8 (14)
C2—C1—C4—C5	−134.2 (6)	C11—C12—C13—C14	−1.4 (13)
N1—C1—C4—C9	−129.2 (6)	C11—C12—C13—Br1	−179.5 (7)
C2—C1—C4—C9	52.3 (9)	C12—C13—C14—C15	2.3 (13)
C9—C4—C5—C6	2.3 (8)	Br1—C13—C14—C15	−179.5 (5)
C1—C4—C5—C6	−171.4 (5)	C13—C14—C15—C10	−1.0 (11)
C4—C5—C6—C7	−0.4 (8)	C11—C10—C15—C14	−1.1 (11)
C5—C6—C7—C8	−1.1 (9)	N2—C10—C15—C14	−179.0 (7)
C5—C6—C7—Cl1	176.4 (4)		

### Methyl (Z)-2-[2-(4-bromophenyl)hydrazin-1-ylidene]-2-(4-chlorophenyl)acetate (5) Hydrogen-bond geometry (Å, º)

**Table:**

*D*—H···*A*	*D*—H	H···*A*	*D*···*A*	*D*—H···*A*
N2—H2···O1	0.93 (5)	2.00 (6)	2.666 (4)	127 (4)
C9—H9···O2	0.95	2.58	2.953 (6)	103
C11—H11···Br1^i^	0.95	2.75	3.689 (4)	172
C12—H12···O1^ii^	0.95	2.54	3.479 (4)	168
C14—H14···Cl1^iii^	0.95	2.70	3.616 (4)	161
C8—H8···*Cg*1^iv^	0.95	2.70	3.591 (6)	157

Symmetry codes: (i) *x*+1/2, −*y*, *z*; (ii) *x*−1/2, −*y*, *z*; (iii) *x*−1/2, −*y*+1, *z*; (iv) −*x*+2, −*y*+1, *z*−1/2.
